# Effectiveness of Personalized Hippocampal Network–Targeted Stimulation in Alzheimer Disease: A Randomized Clinical Trial

**DOI:** 10.1001/jamanetworkopen.2024.9220

**Published:** 2024-05-06

**Authors:** Young Hee Jung, Hyemin Jang, Sungbeen Park, Hee Jin Kim, Sang Won Seo, Guk Bae Kim, Young-Min Shon, Sungshin Kim, Duk L. Na

**Affiliations:** 1 Department of Neurology, Myongji Hospital, Hanyang University, Goyang, Korea; 2 Department of Neurology, Seoul National University Hospital, Seoul National University College of Medicine, Seoul, Korea; 3 Department of Neurology, Samsung Medical Center, Sungkyunkwan University School of Medicine, Samsung Medical Center, Seoul, Korea; 4 Samsung Alzheimer Research Center, Research Institute for Future Medicine, Samsung Medical Center, Seoul, Korea; 5 Department of Artificial Intelligence, Hanyang University, Seoul, Korea; 6 Department of Health Science and Technology, Samsung Advanced Institute for Health Science & Technology, Sungkyunkwan University, Seoul, Korea; 7 Department of Clinical Research Design & Evaluation, Samsung Advanced Institute for Health Science & Technology, Sungkyunkwan University, Seoul, Korea; 8 Anymedi Inc, Seoul, Korea; 9 Smart Healthcare Research Institute, Samsung Medical Center, Seoul, Korea; 10 Department of Data Science, Hanyang University, Seoul, Korea; 11 Center for Neuroscience Imaging Research, Institute for Basic Science, Suwon, Korea; 12 Department of Healthcare Digital Engineering, Hanyang University, Seoul, Korea; 13 Happymind Clinic, Seoul, Korea

## Abstract

**Importance:**

Repetitive transcranial magnetic stimulation (rTMS) has emerged as a safe and promising intervention for Alzheimer disease (AD).

**Objective:**

To investigate the effect of a 4-week personalized hippocampal network–targeted rTMS on cognitive and functional performance, as well as functional connectivity in AD.

**Design, Setting, and Participants:**

This randomized clinical trial, which was sham-controlled and masked to participants and evaluators, was conducted between May 2020 and April 2022 at a single Korean memory clinic. Eligible participants were between ages 55 and 90 years and had confirmed early AD with evidence of an amyloid biomarker. Participants who met the inclusion criteria were randomly assigned to receive hippocampal network–targeted rTMS or sham stimulation. Participants received 4-week rTMS treatment, with assessment conducted at weeks 4 and 8. Data were analyzed between April 2022 and January 2024.

**Interventions:**

Each patient received 20 sessions of personalized rTMS targeting the left parietal area, functionally connected to the hippocampus, based on fMRI connectivity analysis over 4 weeks. The sham group underwent the same procedure, excluding actual magnetic stimulation. A personalized 3-dimensional printed frame to fix the TMS coil to the optimal target site was produced.

**Main Outcomes and Measures:**

The primary outcome was the change in the AD Assessment Scale-Cognitive Subscale test (ADAS-Cog) after 8 weeks from baseline. Secondary outcomes included changes in the Clinical Dementia Rating-Sum of Boxes (CDR-SOB) and Seoul-Instrumental Activity Daily Living (S-IADL) scales, as well as resting-state fMRI connectivity between the hippocampus and cortical areas.

**Results:**

Among 30 participants (18 in the rTMS group; 12 in the sham group) who completed the 8-week trial, the mean (SD) age was 69.8 (9.1) years; 18 (60%) were female. As the primary outcome, the change in ADAS-Cog at the eighth week was significantly different between the rTMS and sham groups (coefficient [SE], −5.2 [1.6]; *P* = .002). The change in CDR-SOB (−4.5 [1.4]; *P* = .007) and S-IADL (1.7 [0.7]; *P* = .004) were significantly different between the groups favoring rTMS groups. The fMRI connectivity analysis revealed that rTMS increased the functional connectivity between the hippocampus and precuneus, with its changes associated with improvements in ADAS-Cog (*r* = −0.57; *P* = .005).

**Conclusions and Relevance:**

This randomized clinical trial demonstrated the positive effects of rTMS on cognitive and functional performance, and the plastic changes in the hippocampal-cortical network. Our results support the consideration of rTMS as a potential treatment for AD.

**Trial Registration:**

ClinicalTrials.gov Identifier: NCT04260724

## Key Points

**Question:**

Can 4-week personalized hippocampal network–targeted stimulation improve cognition in Alzheimer disease?

**Findings:**

In this randomized clinical trial including 30 participants, personalized hippocampal network–targeted stimulation demonstrated a significant improvement in cognition and functional performances compared with the sham group at 8 weeks, with significant improvements evident at 4 weeks after the end of stimulation. Stimulation also increased functional connectivity between the hippocampus and precuneus, with changes correlated with improvements in cognition.

**Meaning:**

These results suggest that personalized hippocampal network–targeted stimulation could be considered as a viable nonpharmacological treatment of Alzheimer disease.

## Teaser

This randomized clinical trial including older Korean adults with Alzheimers disease–related impairment examines cognitive and functional test results for treatment with personalized hippocampal network–targeted stimulation.

## Introduction

Alzheimer disease (AD) is a neurodegenerative disorder characterized by amyloid accumulation in the brain. While only symptomatic treatments have been available, recent approval of antiamyloid monoclonal antibodies has offered potential disease-modifying therapies.^1^ However, given diverse and complex pathological mechanisms, additional therapeutic interventions are still essential for advancing AD management. Repetitive transcranial magnetic stimulation (rTMS) has emerged as a safe potential intervention for AD, with recent clinical trials suggesting improved cognition.^2,3,4,5^

The choice of stimulation site is crucial for rTMS efficacy.^6^ Although the dorsolateral prefrontal cortex (DLPFC) or precuneus have been common target sites,^3,7,8,9,10,11^ the optimal target for rTMS should align with individual disease characteristics and available treatment mechanisms. Early-stage AD has hippocampal atrophy,^12^ leading to functional disconnection with other brain regions, particularly the parietal cortex.^13,14^ Network-targeted rTMS has shown promising results in enhancing interregional functional magnetic resonance image (fMRI) activity within the posterior-medial hippocampal-cortical network.^15,16,17,18,19^ Consequently, rTMS targeting parietal sites could enhance memory performance without directly stimulating the deeply located hippocampus.^15,20^ In addition to selecting stimulation sites, accurate targeting also matters. Personalized stimulation using neuro-navigation systems has been explored to ensure accurate TMS coil positioning.^20^ However, its application in clinical settings can be challenging due to time and resource constraints.

In this study, we conducted a randomized clinical trial to investigate the effectiveness of 4-week personalized hippocampal-network targeted rTMS in AD. Here, we employed personalized fMRI connectivity analysis to guide indirect hippocampal network–targeted stimulation and a personalized 3D-printed frame to secure the coil to the optimal target site. We assessed cognitive and functional performance at the fourth and eighth weeks posttreatment, recruiting patients with AD with confirmed amyloid status. Additionally, we explored the neurobiological basis of cognitive changes, analyzing fMRI-based hippocampal-cortical connectivity.

## Methods

### Study Design and Participants

**Figure 1.**
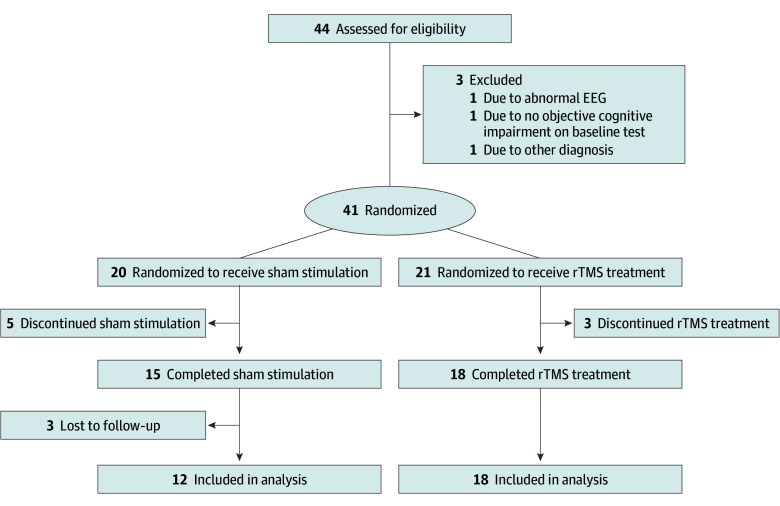
Study Flow Diagram EEG indicates electroencephalogram; rTMS, repetitive transcranial magnetic stimulation.

We conducted a single-centered, randomized, controlled study between May 2020 and April 2022 (NCT04260724) (Figure 1). rTMS (Remed Co Ltd) was given as an add-on to standard treatment, including acetylcholine esterase inhibitors and/or memantine. Eligible participants were randomly assigned in a 1:1 ratio to receive rTMS vs sham stimulation for 4 weeks (eAppendices 1 and 2 in Supplement 1). Cognitive and functional performances were measured at the beginning of the study (baseline, V0) and immediately after 4 weeks of rTMS or sham intervention (V1, 4 weeks from the baseline), and structural MRI and resting-state fMRI were acquired (eAppendices 3 and 4 in Supplement 1). Additionally, after 4 weeks of rTMS stimulation (V2, 8 weeks from the baseline), participants underwent an evaluation of cognitive and functional performances to investigate the long-term effect of rTMS.

Eligible participants were aged between 55 and 90 years with a diagnosis of either mild cognitive impairment due to AD or mild AD dementia based on National Institute on Aging–Alzheimer Association criteria,^21^ with amyloid positivity determined by positron emission tomography (PET)^22,23^ or cerebrospinal fluid (CSF) testing^24^ (eAppendices 5 and 6 in Supplement 1). The trial was approved by the review board and the local ethics committee of Samsung Medical Center. All participants or their legal representatives provided written consent. This study followed the Consolidated Standards of Reporting Trials (CONSORT) reporting guideline. The trial protocol appears in Supplement 2.

### Procedures

**Figure 2.**
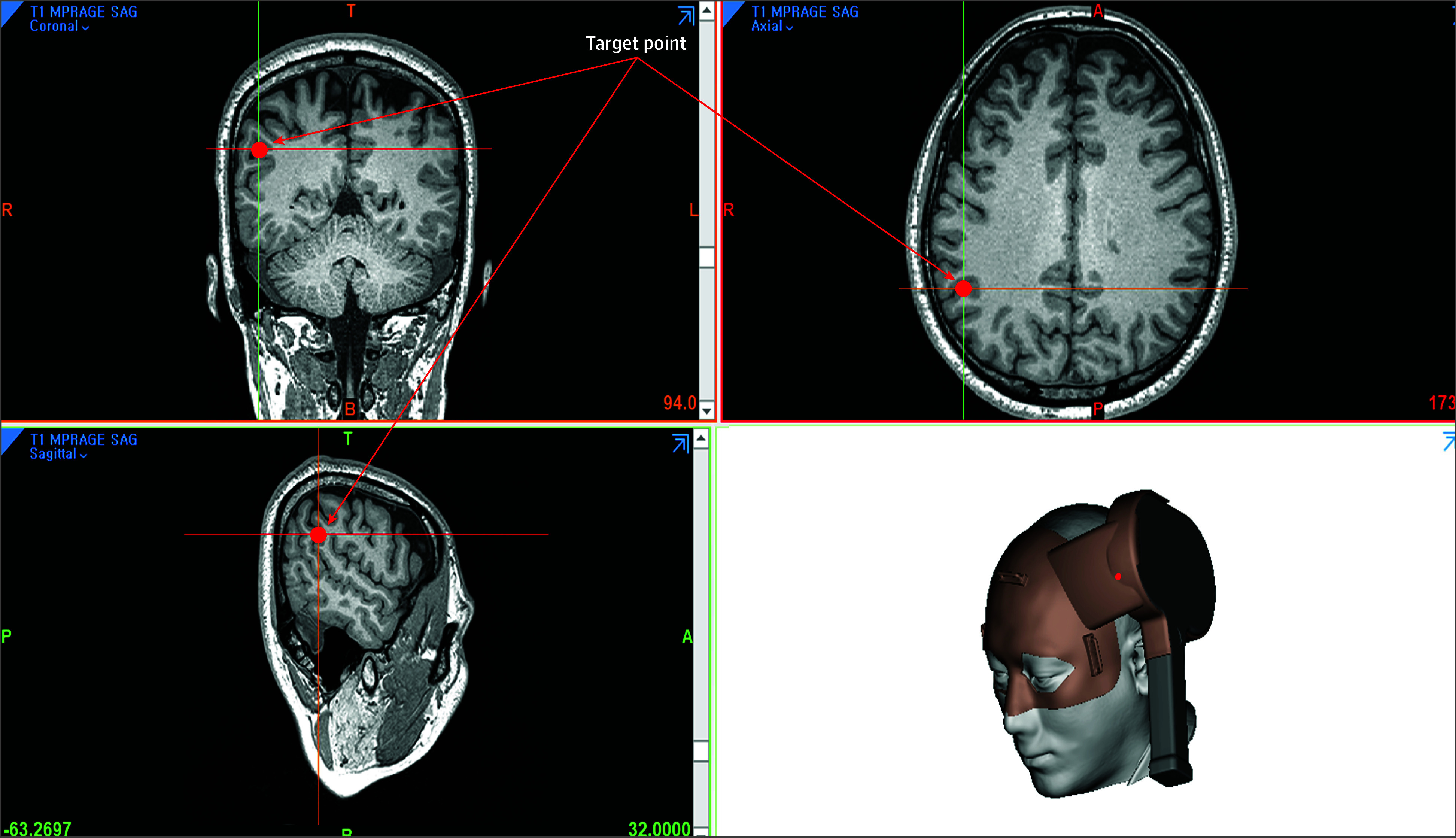
Selection of Target Site and Production of 3-Dimensional (3D) Printed Frame After a target site was determined on the individual T1 structural magnetic resonance image, a personalized 3D-printed frame was produced to fix the transcranial magnetic stimulation coil on the location of the stimulation target.

Stimulation was performed with 40 trains (20 Hz for 2 seconds at 40 pulses/train), which equates to a total of 1600 pulses applied per day and for 5 days per week, following the guidelines on the therapeutic use of rTMS.^8^ The sham group was exposed to the recorded sounds of pulses without real magnetic stimulation. We identified individualized target locations of rTMS from connectivity analysis using resting-state fMRI at baseline, following a procedure based on previous studies.^15,16,19^ Finally, we produced a personalized 3-dimensional (3D) printed frame with space for an rTMS coil of which the center is aligned with the original target coordinate (Anymedi Inc) and used this frame to ensure accurate and consistent targeting of the desired brain region throughout the study (Figure 2; eFigure in Supplement 1). The procedure was safe and well tolerated with no severe adverse events reported. Detailed stimulation procedure with individualized target selection is described in eAppendix 7 in Supplement 1.

### Outcomes

#### Cognitive and Functional Performance

The primary outcome measure was the change of Alzheimer Disease Assessment Scale–Cognitive Subscale-13 (ADAS-Cog-13) at 8 weeks from baseline. The secondary outcomes were the change of the ADAS-Cog-13 at 4 weeks from baseline. Other secondary outcomes included the change at 4 and 8 weeks from baseline of controlled oral word association test (COWAT), Stroop Color Test, and trail making test (TMT),^25,26^ Korean versions of the Mini-Mental Status Examination (MMSE),^27^ Clinical deterioration rating sum of boxes (CDR-SOB),^28^ Seoul-Instrumental Activities of Daily Living (S-IADL),^29^ Korean version of the Montreal Cognitive assessment (MOCA-K), and 7 subsets of the computer-based Cambridge Neuropsychological Test Automated Battery (CANTAB; Cambridge Cognition).^30^

#### Neuroimaging

We measured changes in the hippocampal-cortical functional connectivity after 4 weeks using resting-state fMRI. Specifically, we first defined the precuneus as a region of interest (ROI), a core region of the posterior medial hippocampal network, where we predicted an enhancement in connectivity with the hippocampus responding to rTMS.^15,16,17,18,19^ Then, we further identified a significant rTMS-responsive region within the ROI and correlated the changes in its connectivity with the hippocampus due to rTMS with changes in ADAS-Cog score, which was our clinical outcome. We compared the neuroimaging outcomes between the rTMS group and the sham group to validate the efficacy of the rTMS treatment (eAppendix 8 in Supplement 1).

### Statistical Analyses

The sample size was estimated based on a previous study^31^ (eAppendix 9 in Supplement 1). Clinical characteristics between the 2 groups were compared using a 2-sample *t* test or a χ^2^ test. We used linear mixed model analysis to investigate the effect of intervention, with fixed effects including age, sex, education years, apolipoprotein E (APOE) ε4 carrier status, group (rTMS vs sham), visit (V0, V1, and V2), and group-by-visit interaction term. Race and ethnicity were not included because the ethnicity of the participants was homogeneous, comprising solely individuals of Korean descent. We used a random-effects model to explain repeated measures within patients. Statistical significance was indicated by *P* ≤ .05 in 2-sided tests. All analyses for cognitive and functional scores were performed using R version 4.3.2 and R studio version 2023.12.0 + 369 (R Project for Statistical Computing).

The significance of the neuroimaging outcome, hippocampal-cortical connectivity, was corrected for multiple *t* tests at a cluster level within the predefined precuneus ROI (3808 voxels) using AFNI software version 23.1.01 (National Institutes of Health).^32^ Monte Carlo simulation performed within the precuneus ROI determined a spatial extent threshold of 195 contiguous voxels with a cluster-wise *P* < .05 (eAppendix 8 in Supplement 1). In the subsequent analysis, we correlated the averaged connectivity changes within the cluster from the baseline to the fourth week posttreatment (V1) with the changes in ADAS-Cog scores. The association between the changes in connectivity and those in ADAS-Cog was assessed as a Spearman rank correlation due to the limited sample size (eAppendix 8 in Supplement 1).

## Results

### Study Population, Adherence to Intervention, and Background Characteristics

Of the 44 participants screened between May 2020 and Dec 2021, 41 were randomly assigned. Eleven participants withdrew or were lost to follow-up for 8 weeks. Among 30 participants (18 in the rTMS group; 12 in the sham group) who completed the 8-week trial, the mean (SD) age was 69.8 (9.1) years; 18 participants (60%) were female (eTable 1 in Supplement 1).

### Changes in Cognitive Performance

**Figure 3.**
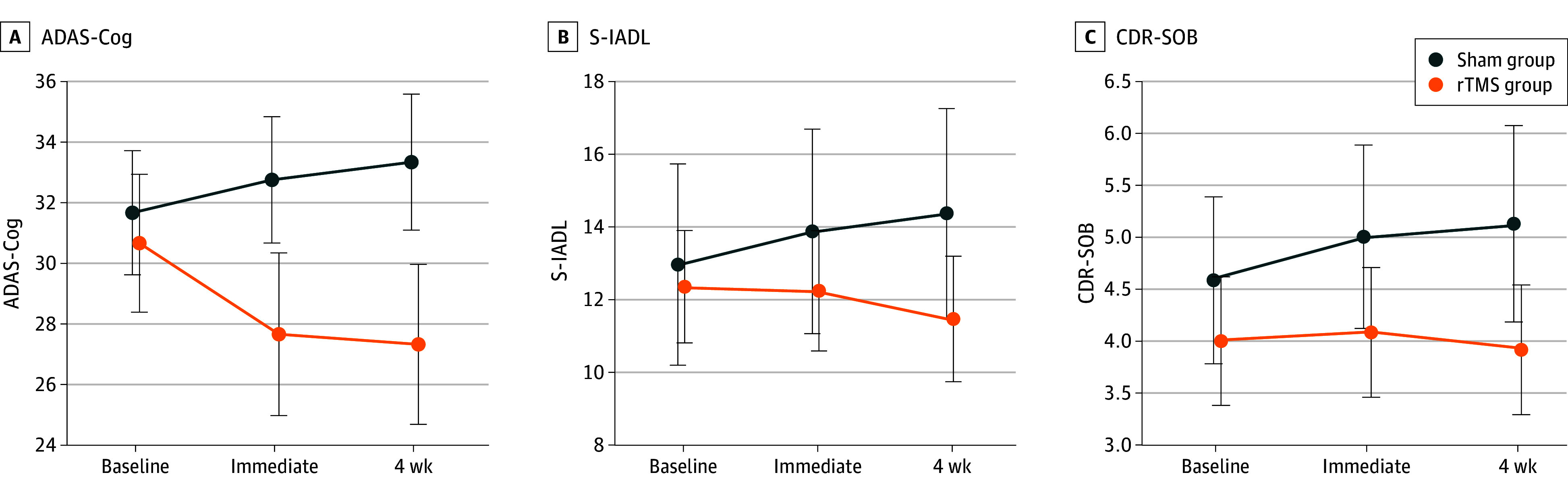
Cognitive Outcomes After Repetitive Transcranial Magnetic Stimulation (rTMS) Treatment ADAS-Cog indicates Alzheimer Disease Assessment Scale-Cognitive Subscale; CDR-SOB, clinical deterioration rating sum of boxes; S-IADL, Seoul-Instrumental Activities of Daily Living. Time points include 3 test sessions: pretreatment, immediately after 4-week rTMS treatment, and an additional 4 weeks after the rTMS treatment. A lower score means improvement, with error bars indicating the standard of error of the mean.

As a primary outcome, the group-by-visit interaction effect on ADAS-Cog was significant at V2 (eighth week) from baseline (coefficient [SE], −5.2 [1.6]; *P* = .002), which demonstrated that the rTMS group showed significant improvement in ADAS-Cog compared with the sham group. The difference in improvement between the groups was also significant at the fourth week (V1, −4.4 [1.6]; *P* = .007) (Figure 3 and Table). The pairwise post hoc comparisons demonstrated that ADAS-Cog significantly improved in the rTMS group at both V1 (3.24 [0.95]; *P* = .01) and V2 (3.53 [1.01]; *P* = .01) time points (eTable 2 in Supplement 1).

**Table.** Effect of the Hippocampal Network–Targeted Repetitive Transcranial Magnetic Stimulation (rTMS) on Cognitive and Functional Performances

Scale	Sham, mean (SD) (n = 12)^a^			rTMS, mean (SD) (n = 18)^a^			Intervention effect (group × visit)			
	V0 (0 wk)	V1 (4 wk)	V2 (8 wk)	V0 (0 wk)	V1 (4 wk)	V2 (8 wk)	V1 (4 wk)		V2 (8 wk)	
							Coefficient (SE)	*P* value	Coefficient (SE)	*P* value
ADAS-Cog total	31.7 (7.1)	32.8 (7.2)	33.3 (7.6)	30.7 (9.6)	27.7 (11.4)	27.3 (11.2)	−4.4 (1.6)	.007	−5.2 (1.6)	.002
Memory	27.1 (5.6)	28.2 (5.7)	28.1 (4.8)	26.0 (6.5)	23.6 (7.2)	22.5 (7.8)	−3.5 (1.4)	.01	−4.5 (1.4)	.002
Language	1.4 (2.0)	1.1 (1.7)	0.4 (1.2)	1.1 (2.2)	1.2 (3.1)	1.7 (4.2)	0.4 (0.7)	.55	1.7 (0.7)	.03
Praxis	1.6 (1.9)	2.0 (1.6)	1.4 (1.2)	1.8 (1.5)	1.6 (1.9)	1.7 (2.1)	−0.6 (0.7)	.33	−2.097 × 10^−15^ (0.7)	>.99
MMSE	22.5 (3.6)	22.5 (3.6)	22.4 (3.4)	23.9 (3.5)	24.4 (3.0)	24.4 (3.4)	0.6 (0.8)	.44	0.5 (0.8)	.51
MOCA	19.7 (2.3)	18.8 (3.4)	19.3 (3.9)	19.9 (4.3)	20.4 (4.4)	19.7 (6.3)	1.3 (1.2)	.27	0.3 (1.2)	.78
CDR-SOB	4.6 (2.8)	5.0 (3.0)	5.1 (3.3)	4.0 (2.6)	4.1 (2.6)	3.9 (2.6)	−0.6 (0.3)	.05	−0.78 (0.3)	.008
SIADL	13.0 (9.6)	13.9 (9.8)	14.4 (10.0)	12.4 (6.6)	12.3 (7.0)	11.5 (7.4)	−1.1 (0.7)	.16	−2.4 (0.8)	.002
Frontal-executive tests										
COWAT-semantic	13.4 (6.9)	10.7 (4.8)	11.3 (5.0)	12.9 (5.6)	13.2 (5.3)	13.8 (5.2)	−0.5 (1.9)	.80	0.6 (1.9)	.76
COWAT-phonemic	8.1 (3.7)	8.2 (3.8)	8.3 (3.9)	10.6 (6.0)	10.6 (5.8)	10.0 (5.4)	−0.7 (2.1)	.75	−2.3 (2.2)	.30
K-TMT-B	92.2 (72.2)	96.8 (71.8)	110.6 (100.5)	91.2 (98.2)	94.6 (102.7)	88.9 (100.4)	−3.7 (15.3)	.81	−21.8 (15.6)	.17
Stroop color	64.7 (27.2)	56.8 (31.3)	62.0 (26.2)	81.4 (28.0)	83.3 (30.3)	85.9 (29.9)	9.0 (4.8)	.06	7.2 (4.9)	.15
CANTAB										
DMS	56.7 (15.3)	63.2 (16.3)	57.2 (15.6)	66.7 (14.4)	65.6 (15.9)	63.4 (20.7)	−5.9 (6.2)	.35	−3.1 (6.5)	.64
MOT	958.9 (294.0)	927.1 (186.3)	875.2 (136.6)	886.5 (215.3)	852.0 (207.0)	852.0 (207.0)	4.8 (70.1)	.95	−3.9 (72.8)	.94
PAL	60.0 (8.4)	57.9 (8.8)	57.9 (8.8)	53.3 (13.7)	55.0 (10.8)	56.7 (9.0)	4.4 (2.8)	.12	5.6 (2.9)	.06
PRM	72.2 (12.5)	72.2 (12.5)	59.0 (13.5)	73.6 (15.7)	73.6 (15.7)	68.1 (22.9)	5.9 (7.4)	.43	10.5 (7.6)	.18
RTI	433.7 (56.0)	420.7 (67.5)	423.4 (67.8)	389.2 (45.4)	377.3 (40.6)	389.6 (43.9)	−3.8 (10.6)	.72	6.1 (11.0)	.58
RVP	668.6 (87.6)	727.3 (187.2)	663.7 (137.8)	698.7 (225.0)	556.0 (138.0)	563.7 (177.4)	−0.001 (0.003)	.68	−0.005 (0.003)	.86
SWM	10.3 (1.1)	9.8 (1.0)	9.5 (1.1)	9.4 (1.3)	9.5 (1.1)	9.6 (1.2)	0.5 (0.5)	.41	0.9 (0.6)	.12

Abbreviations: ADAS-Cog, Alzheimer Disease Assessment Scale-Cognitive Subscale; CANTAB, Cambridge Neuropsychological Test Automated Battery; CDR-SOB, clinical dementia rating-sum of boxes; COWAT, controlled oral word association test; DMS, delayed match to sample; K-TMT-B, Korean version–trail making test-part B; MOCA, Montreal Cognitive Assessment; MOT, motor screening task; PAL, paired associates learning; PRM, pattern recognition memory; RTI, reaction time task; RVP, rapid visual information processing; S-IADL, Seoul–Instrumental Activity of Daily Living; SWM, spatial working memory; V, visit.

^a^ Adjusted for age, sex, education year, and apolipoprotein E genotype.

We found no significant differences in intervention effects based on cognitive status (aMCI vs ADD) (eAppendix 10 in Supplement 1). We additionally categorized ADAS-Cog scores into 3 domain-specific scores: memory, language, and praxis.^33^ The group-by-visit interaction effect at V2 from V0 was significant only in the memory domain (−4.5 [1.4]; *P* = .002). Although this interaction effect was significant at V2 in the language domain (1.7 [0.7]; *P* = .03), the direction was reversed, and we did not see a significant group-by-visit interaction effect in the praxis domain (−2.097 × 10^−15^ [0.7]; *P* = .97) (Table).

The rTMS group showed a larger improvement in CDR-SOB than the sham group at V2 (−0.78 [0.3]; *P* = .008), and the difference in CDR-SOB change between the groups was also significant at V1 (0.6 [0.3]; *P* = .046). Also, the rTMS group showed a larger improvement in S-IADL than the sham group at V2 (−2.4 [0.8]; *P* = .16), while the difference in S-IADL change between the groups was not significant at V1 (*P* = .002) (Figure 3; Table). There was no significant difference between the groups both at V1 and V2 in secondary outcomes, including MMSE, MOCA-K, and all components in frontal-executive tests and CANTAB (Table).

### Changes in Functional Connectivity

**Figure 4.**
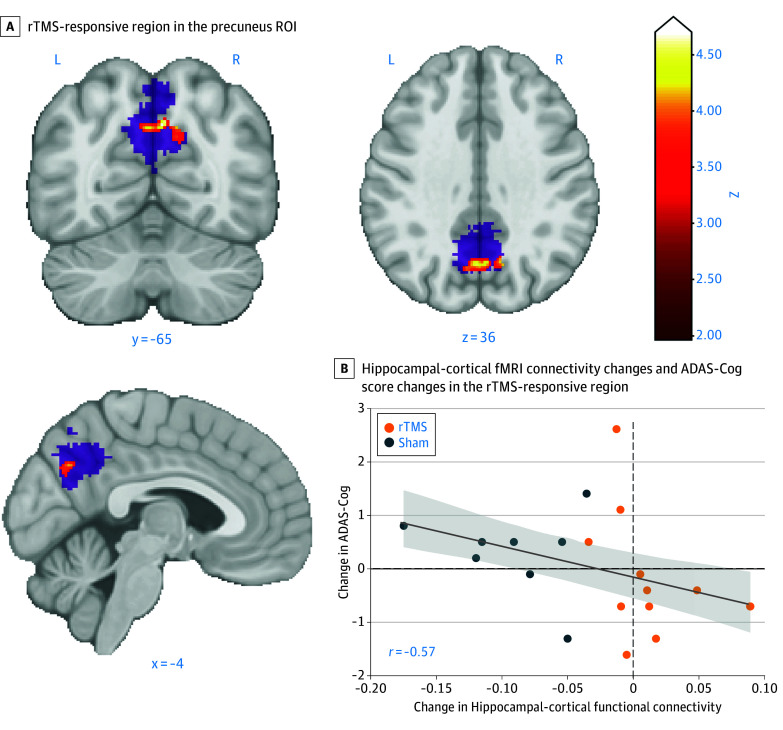
Neuroimaging Outcomes A, Repetitive transcranial magnetic stimulation (rTMS)-responsive region in the precuneus region of interest (ROI) (purple) shows larger connectivity changes from the baseline to post-rTMS treatment relative to the sham group. B, Association between the hippocampal-cortical functional magnetic resonance imaging (fMRI) connectivity changes and Alzheimer Disease Assessment Scale-Cognitive Subscale (ADAS-Cog) score changes in the rTMS-responsive region (cluster in the purple region in C).

For the fMRI connectivity analysis, 8 patients were excluded (4 patients from each of the rTMS and sham groups) because MRI acquisition protocols differed between pre- and posttreatment due to operator errors. Subsequently, 2 participants from the rTMS group failed to complete the posttreatment fMRI sessions, which led to a final sample size of 20 (rTMS, 12 patients; sham, 8 patients). Patient-specific rTMS targets in the left parietal regions, defined based on seed-based resting-state connectivity analysis, are available in eTable 3 in Supplement 1. Within the predefined precuneus ROI, we searched for rTMS-responsive regions, which showed increased connectivity with the hippocampal seeds due to rTMS relative to sham (Figure 4A). We identified 2 main clusters of 228 (770 mm^3^) and 174 (587 mm^3^) supra-subthreshold voxels within the ROI, comparable with previous TMS studies.^3,10,15,16^ Only the larger cluster remained significant after small-volume correction using the predefined precuneus ROI (eAppendix 8 in Supplement 1).

Thus, in the subsequent analysis, we correlated the averaged connectivity changes within the cluster from the baseline to the fourth week (V1) with the changes in ADAS-Cog scores. We found that change in ADAS-Cog significantly correlated with corresponding changes in connectivity between the hippocampal seed and the identified rTMS-responsive region (*r* = −0.57; *P* = .005 in a 1-sided test with a hypothesis of hippocampal-cortical connectivity). The association was also significant for the rTMS group only (12 patients) (*r* = −0.56; *P* = .03) but not significant for the sham group only (8 patients) (*r* = −0.07; *P* = .43) (Figure 4B). Notably, we defined the rTMS-responsive region relative to sham, and thus, the rTMS did not necessarily increase the connectivity for the rTMS group after treatment. Indeed, we found no significant increase (*P* = .13 in a 1-sided Wilcoxon signed-rank test) from the baseline to V2. The weakened rTMS effects on the connectivity change could be due to the significant decline of the connectivity in patients with AD, as shown in the sham group (2-tailed Wilcoxon signed-rank test, *P* = .008). Our results suggest that the hippocampal-cortical connectivity would naturally decline as a progression of AD^10,34^ without rTMS treatment. Despite a small sample size, the result demonstrated that the increase in hippocampal-cortical connectivity in the precuneus caused by rTMS provides a reliable estimate of improvement, evident in the decrease in ADAS-Cog scores (Figure 4B).

## Discussion

In this randomized clinical trial, we investigated the effectiveness of 4-week personalized hippocampal network–targeted rTMS for treatment in AD. Our first significant finding was that rTMS improved cognitive and functional performances, particularly at the eighth week, but improvements were evident as soon as 4 weeks after the end of rTMS. The second significant finding was that the improvement in ADAS-Cog scores was significantly correlated with the increased functional connectivity between the hippocampus and the precuneus after rTMS, as determined through resting-state fMRI data analysis. The positive results of our study suggest that rTMS could be considered an add-on treatment for AD.

The first significant finding was improved cognitive and functional performance after hippocampal network targeted stimulation. We found a positive rTMS effect on ADAS-Cog, particularly in the memory domain, as we intended to target the hippocampal memory network. We also found a significant effect of rTMS on improving S-IADL and CDR-SOB, which contain aspects of functional performance,^36^ compared with the sham group at V2. If the rTMS can aid patients with AD in maintaining functional performance, it could delay the progression to severe dementia and help alleviate caregiver burdens. Interestingly, there were no significant effects on S-IADL and CDR-SOB at V1. It may suggest that rTMS might affect cortical plasticity as a long-term change in AD, which is consistent with previous studies.^37,38,39,40^ In fact, patients with AD are known to have dramatically impaired long-term potentiation (LTP), particularly in the hippocampal CA1 region, and facilitated long-term depression.^41^ In our study, hippocampal network–targeted rTMS may modulate synaptic plasticity, alleviating memory deficits by facilitating LTP. As an underlying mechanism, previous studies showed that rTMS increased the expression of the dopamine DR4 gene and brain-derived neurotrophic factor (BDNF) in the cerebral cortex and hippocampus in AD mouse models.^42,43^ It has also been shown that the modulatory effect of rTMS is reduced in participants carrying the Val66Met allele of the BDNF gene.^44^ Moreover, it was suggested that rTMS could counteract mechanisms of apoptosis, leading to decreased phosphorylated tau and Aβ expression.^37,42,45^ However, further studies with an assessment of relevant biomarkers are required to understand the underlying mechanism of the long-term effect of rTMS on cognitive and functional performance.

We could not prove the effect of rTMS on MMSE, MOCA, frontal-executive function, and cognitive outcome measures in CANTAB. Although MMSE and MOCA are commonly used as initial screening tools for dementia, they are less sensitive to detect longitudinal cognitive change, particularly in an earlier stage of AD,^35^ compared with ADAS-Cog (which covers a broader range of cognitive domains) or CDR-SOB (which comprehensively assesses both cognition and functional abilities). Regarding the Paired Associated Learning (PAL) task in CANTAB, aimed mainly at evaluating memory function, we did not observe a significant improvement, unlike previous studies in cognitively unimpaired or younger adults.^15,46^ This difference could be attributed to variations in study protocols, age demographics, or unfamiliarity with computerized tasks among our cohort of older Korean adults. Therefore, the CANTAB memory tasks might not have effectively assessed memory function. Although the rTMS effect was also significant in the language domain of ADAS-Cog, the direction was reversed as the sham group showed an improved score. The result might be attributed to the ceiling effects in language domain score changes because the mean language score of our study participants was generally low.

The second significant finding was that hippocampal network–targeted rTMS increased functional connectivity between the precuneus and the hippocampus compared with the sham control. We also found that the increased connectivity correlated with improved cognitive scores measured as ADAS-Cog. As a core region in the default mode network (DMN), the precuneus is critically involved in episodic memory retrieval.^47^ Its abnormal activity with reduced functional connectivity is considered a biomarker of early AD.^10,34^ Thus, our neuroimaging results aligning with the critical role of the precuneus are noteworthy because the measurable changes in the precuneus supported the improvement in clinical scores. These changes could serve as a biomarker for treatment effectiveness, further strengthening the credibility of our network-targeting rTMS approach to modulate the hippocampal-cortical memory network. As we hypothesized, this network change might be attributed to facilitating LTP-like plastic changes. Nevertheless, whether this mechanism effectively leads to structural changes encompassing long-term neural regeneration remains uncertain.

As a personalized stimulation site for rTMS in patients with AD, we determined the left parietal area based on previous studies.^7,46,48,49,50^ In fact, most early rTMS studies used DLPFC as the target site given its involvement in mood regulation and cognitive control based on rTMS use for depression treatment,^7,8,9^ and the personalized stimulation of DLPFC also has been successful for depression treatment (ie, the SAINT protocol).^51^ A 2022 trial suggested that precuneus could be one of the beneficial rTMS targets considering DMN,^3,10^ but comprehensive studies on precuneus-rTMS are limited.^3,10,11^ Our stimulation selection was based on the knowledge that rTMS can increase fMRI activity across the hippocampal-cortical network in DMN^15,16,17,18,19^ by stimulating the lateral parietal region, which is functionally connected to the hippocampus.^40^ The parietal cortex has projections to the retrosplenial and parahippocampal cortices, which also provide input to the hippocampus.^13,14^ This has significant clinical implications because direct access to the hippocampus, located deep within the brain, is challenging through TMS.^15,20^ Particularly, our study’s strength lies in the personalized fMRI-guided approach for this stimulation, given that a similar fMRI-guided rTMS protocol for depression treatment achieved higher remission rates than conventional protocols.^51^ In future studies, comparing effects based on different target sites or stimulation of multiple sites will be crucial for determining the optimal target selection of rTMS for AD treatment.

Another important issue in rTMS application as therapeutics is the need to accurately and consistently stimulate the same target site within and across sessions.^6^ Traditional studies used gross anatomical landmarks (eg, the 5 cm rule for DLPFC identification) to choose the stimulation site. Recent research has explored participant-specific stimulation using neuroimaging scans, leading to improved outcomes.^3,6,15,19^ However, using neuro-navigation systems for each stimulation remains challenging due to time, training, space, and cost requirements. We utilized a personalized 3D-printed frame, presenting a practical alternative to neuro-navigation systems for accurate coil positioning. In addition, it is noteworthy that our study recruited participants with evidence of amyloid positivity on PET scans or CSF testing, which is the core biomarker for AD, to minimize the inclusion of participants with cognitive impairments due to non-Alzheimer pathophysiologies.

### Limitations

There are several limitations in our study. First, the sample size was relatively small, and more study participants were unequally lost to follow-up during the trial, leading to unbalanced study participants between the rTMS and sham groups. Second, as the sham coil only recreated the sound of an active coil without magnetic stimulation, nonspecific effects of TMS, such as peripheral nerve or muscle stimulation and bone conduction, could not be ruled out. Third, although we recruited patients with amyloid biomarkers, they cannot be considered AD without evidence of tau deposition.

## Conclusions

In our randomized clinical trial, personalized hippocampal network–targeted stimulation demonstrated positive effects of rTMS on cognition, including improved performance in cognition and functional tests compared with a sham group. Combined with the observed plastic changes in the hippocampal-cortical network, our results support the consideration of rTMS as a potential nonpharmacological treatment for AD.

## Notes

Supplement 1. **eAppendix 1.** Eligibility Criteria **eAppendix 2.** Randomization and Masking **eAppendix 3.** Brain MRI Acquisition **eAppendix 4.** MRI Data Preprocessing **eAppendix 5.** Aβ PET Acquisition and Definition of Aβ Positivity **eAppendix 6.** CSF Collection and AD Biomarker Analysis **eAppendix 7.** Stimulation Procedure With Target Selection Based on fMRI Data Analysis **eAppendix 8.** fMRI Data Analysis **eAppendix 9.** Sample Size **eTable 1.** Characteristics of Study Participants Who Completed the Trial **eTable 2.** Paired Comparison of the Neuropsychological Profiles of Each Visit Across the Groups **eTable 3.** Original and MNI Coordinates for Individual Patients **eFigure.** Modeling for a Personalized 3D-Printed Frame as a TMS Guide **eAppendix 10.** Effect of the Hippocampal Network–Targeted rTMS on ADAS-Cog Change According to the Diagnosis
eReferences

Supplement 2.
Trial Protocol

Supplement 3.
Data Sharing Statement

## References

[1] van Dyck CH, Swanson CJ, Aisen P, . Lecanemab in early Alzheimer’s disease. N Engl J Med. 2023;388(1):9-21. doi:10.1056/NEJMoa2212948 36449413

[2] Chou Y-H, That VT, Sundman M. A systematic review and meta-analysis of rTMS effects on cognitive enhancement in mild cognitive impairment and Alzheimer’s disease. Neurobiol Aging. 2020;86:1-10. doi:10.1016/j.neurobiolaging.2019.08.020 31783330 PMC6995441

[3] Koch G, Casula EP, Bonnì S, . Precuneus magnetic stimulation for Alzheimer’s disease: a randomized, sham-controlled trial. Brain. 2022;145(11):3776-3786. doi:10.1093/brain/awac285 36281767 PMC9679166

[4] Cotelli M, Manenti R, Cappa SF, . Effect of transcranial magnetic stimulation on action naming in patients with Alzheimer disease. Arch Neurol. 2006;63(11):1602-1604. doi:10.1001/archneur.63.11.1602 17101829

[5] Cotelli M, Manenti R, Cappa S, Zanetti O, Miniussi C. Transcranial magnetic stimulation improves naming in Alzheimer disease patients at different stages of cognitive decline. Eur J Neurol. 2008;15(12):1286-1292. doi:10.1111/j.1468-1331.2008.02202.x 19049544

[6] Menardi A, Dotti L, Ambrosini E, Vallesi A. Transcranial magnetic stimulation treatment in Alzheimer’s disease: a meta-analysis of its efficacy as a function of protocol characteristics and degree of personalization. J Neurol. 2022;269(10):5283-5301. doi:10.1007/s00415-022-11236-2 35781536 PMC9468063

[7] Wang X, Mao Z, Ling Z, Yu X. Repetitive transcranial magnetic stimulation for cognitive impairment in Alzheimer’s disease: a meta-analysis of randomized controlled trials. J Neurol. 2020;267(3):791-801. doi:10.1007/s00415-019-09644-y 31760522

[8] Lefaucheur J-P, André-Obadia N, Antal A, . Evidence-based guidelines on the therapeutic use of repetitive transcranial magnetic stimulation (rTMS). Clin Neurophysiol. 2014;125(11):2150-2206. doi:10.1016/j.clinph.2014.05.021 25034472

[9] Lefaucheur J-P, Aleman A, Baeken C, . Evidence-based guidelines on the therapeutic use of repetitive transcranial magnetic stimulation (rTMS): an update (2014–2018). Clin Neurophysiol. 2020;131(2):474-528. doi:10.1016/j.clinph.2019.11.002 31901449

[10] Koch G, Bonnì S, Pellicciari MC, . Transcranial magnetic stimulation of the precuneus enhances memory and neural activity in prodromal Alzheimer’s disease. Neuroimage. 2018;169:302-311. doi:10.1016/j.neuroimage.2017.12.048 29277405

[11] Bonnì S, Veniero D, Mastropasqua C, . TMS evidence for a selective role of the precuneus in source memory retrieval. Behav Brain Res. 2015;282:70-75. doi:10.1016/j.bbr.2014.12.032 25541040

[12] Rao YL, Ganaraja B, Murlimanju B, Joy T, Krishnamurthy A, Agrawal AJB. Hippocampus and its involvement in Alzheimer’s disease: a review. 3 Biotech. 2022;12(2):55. doi:10.1007/s13205-022-03123-4 PMC8807768 35116217

[13] Mesulam M-M, Van Hoesen GW, Pandya DN, Geschwind N. Limbic and sensory connections of the inferior parietal lobule (area PG) in the rhesus monkey: a study with a new method for horseradish peroxidase histochemistry. Brain Res. 1977;136(3):393-414. doi:10.1016/0006-8993(77)90066-x 411543

[14] Mufson EJ, Pandya DN. Some observations on the course and composition of the cingulum bundle in the rhesus monkey. J Comp Neurol. 1984;225(1):31-43. doi:10.1002/cne.902250105 6725639

[15] Wang JX, Rogers LM, Gross EZ, . Targeted enhancement of cortical-hippocampal brain networks and associative memory. Science. 2014;345(6200):1054-1057. doi:10.1126/science.1252900 25170153 PMC4307924

[16] Kim S, Nilakantan AS, Hermiller MS, Palumbo RT, VanHaerents S, Voss JL. Selective and coherent activity increases due to stimulation indicate functional distinctions between episodic memory networks. Sci Adv. 2018;4(8):eaar2768. doi:10.1126/sciadv.aar2768 30140737 PMC6105230

[17] Warren KN, Hermiller MS, Nilakantan AS, Voss JLJE. Stimulating the hippocampal posterior-medial network enhances task-dependent connectivity and memory. eLife. 2019;8:e49458. doi:10.7554/eLife.49458 31724946 PMC6855798

[18] Freedberg M, Cunningham CA, Fioriti CM, . Multiple parietal pathways are associated with rTMS-induced hippocampal network enhancement and episodic memory changes. Neuroimage. 2021;237:118199. doi:10.1016/j.neuroimage.2021.118199 34033914 PMC8926059

[19] Nilakantan AS, Mesulam M-M, Weintraub S, Karp EL, VanHaerents S, Voss JLJN. Network-targeted stimulation engages neurobehavioral hallmarks of age-related memory decline. Neurology. 2019;92(20):e2349-e2354. doi:10.1212/WNL.0000000000007502 30996057 PMC6598819

[20] Cash RF, Hendrikse J, Fernando KB, . Personalized brain stimulation of memory networks. Brain Stimul. 2022;15(5):1300-1304. doi:10.1016/j.brs.2022.09.004 36113762

[21] McKhann GM, Knopman DS, Chertkow H, . The diagnosis of dementia due to Alzheimer’s disease: Recommendations from the National Institute on Aging-Alzheimer’s Association workgroups on diagnostic guidelines for Alzheimer’s disease. Alzheimers Dement. 2011;7(3):263-269. doi:10.1016/j.jalz.2011.03.005 21514250 PMC3312024

[22] Barthel H, Gertz H-J, Dresel S, . Cerebral amyloid-β PET with florbetaben (18F) in patients with Alzheimer’s disease and healthy controls: a multicentre phase 2 diagnostic study. Lancet Neurol. 2011;10(5):424-435. doi:10.1016/S1474-4422(11)70077-1 21481640

[23] Farrar G, Molinuevo JL, Zanette M. Is there a difference in regional read [18 F] flutemetamol amyloid patterns between end-of-life subjects and those with amnestic mild cognitive impairment? Eur J Nucl Med Mol Imaging. 2019;46(6):1299-1308. doi:10.1007/s00259-019-04282-y 30863934 PMC6486895

[24] Lee J, Jang H, Kang SH, . Cerebrospinal fluid biomarkers for the diagnosis and classification of Alzheimer’s disease spectrum. J Korean Med Sci. 2020;35(44):e361. doi:10.3346/jkms.2020.35.e361 33200589 PMC7669457

[25] Kang SH, Park YH, Lee D, . The cortical neuroanatomy related to specific neuropsychological deficits in Alzheimer’s continuum. Dement Neurocogn Disord. 2019;18(3):77-95. doi:10.12779/dnd.2019.18.3.77 31681443 PMC6819670

[26] Kang Y, Na D, Hahn S. Seoul Neuropsychological Screening Battery. Incheon: Human Brain Research & Consulting Co; 2003.

[27] Kang Y, Na D-L, Hahn SH. A validity study on the Korean Mini-Mental State Examination (K-MMSE) in dementia patients. J Korean Neurol Asso. 1997;15(2):300-308.

[28] Morris JC. The clinical dementia rating (CDR): Current version and scoring rules. Neurology. 1993;32(11):2412-2414. doi:10.1212/wnl.43.11.2412-a 8232972

[29] Ku H-M, Kim J-H, Kwon E-J, . A study on the reliability and validity of Seoul-Instrumental Activities of Daily Living. J Korean Geriatric Soc. 2004;8(4):206-214.

[30] Égerházi A, Berecz R, Bartók E, Degrell I. Automated Neuropsychological Test Battery (CANTAB) in mild cognitive impairment and in Alzheimer’s disease. Prog Neuropsychopharmacol Biol Psychiatry. 2007;31(3):746-751. doi:10.1016/j.pnpbp.2007.01.011 17289240

[31] Rabey JM, Dobronevsky E, Aichenbaum S, Gonen O, Marton RG, Khaigrekht M. Repetitive transcranial magnetic stimulation combined with cognitive training is a safe and effective modality for the treatment of Alzheimer’s disease: a randomized, double-blind study. Neurol Preclinical Neurological Stud. 2013;120(5):813-819. doi:10.1007/s00702-012-0902-z 23076723

[32] Cox RW. AFNI: software for analysis and visualization of functional magnetic resonance neuroimages. Comput Biomed Res. 1996;29(3):162-173. doi:10.1006/cbmr.1996.0014 8812068

[33] Cogo-Moreira H, Krance SH, Wu CY, ; Alzheimer’s Disease Neuroimaging Initiative. State, trait, and accumulated features of the Alzheimer’s Disease Assessment Scale Cognitive Subscale (ADAS-Cog) in mild Alzheimer’s disease. Alzheimers Dement (N Y). 2023;9(1):e12376. doi:10.1002/trc2.12376 36994227 PMC10040491

[34] Chen Y, Liu Z, Zhang J, . Precuneus degeneration in nondemented elderly individuals with APOE ε4: evidence from structural and functional MRI analyses. Hum Brain Mapp. 2017;38(1):271-282. doi:10.1002/hbm.23359 27593520 PMC6866889

[35] Mohs RC, Knopman D, Petersen RC, . Development of cognitive instruments for use in clinical trials of antidementia drugs: additions to the Alzheimer’s Disease Assessment Scale that broaden its scope. Alzheimer Dis Assoc Disord. 1997;11(Suppl_2):S13-S21. 9236948

[36] Akpınar Söylemez B, Küçükgüçlü Ö, Akyol MA, Işık AT. Quality of life and factors affecting it in patients with Alzheimer’s disease: a cross-sectional study. Health Qual Life Outcomes. 2020;18(1):304. doi:10.1186/s12955-020-01554-2 32912233 PMC7488137

[37] Cirillo G, Di Pino G, Capone F, . Neurobiological after-effects of non-invasive brain stimulation. Brain Stimul. 2017;10(1):1-18. doi:10.1016/j.brs.2016.11.009 27931886

[38] Li X, Qi G, Yu C, . Cortical plasticity is correlated with cognitive improvement in Alzheimer’s disease patients after rTMS treatment. Brain Stimul. 2021;14(3):503-510. doi:10.1016/j.brs.2021.01.012 33581283

[39] Alcalá-Lozano R, Morelos-Santana E, Cortes-Sotres JF, Garza-Villarreal EA, Sosa-Ortiz A, Gonzalez-Olvera JJ. Similar clinical improvement and maintenance after rTMS at 5 Hz using a simple vs. complex protocol in Alzheimer’s disease. Brain Stimul. 2018;11(3):625-627. doi:10.1016/j.brs.2017.12.011 29326021

[40] Zhang F, Qin Y, Xie L, Zheng C, Huang X, Zhang M. High-frequency repetitive transcranial magnetic stimulation combined with cognitive training improves cognitive function and cortical metabolic ratios in Alzheimer’s disease. J Neural Transm. 2019;126(8):1081-1094. doi:10.1007/s00702-019-02022-y 31292734

[41] Koch G, Di Lorenzo F, Bonnì S, Ponzo V, Caltagirone C, Martorana A. Impaired LTP- but not LTD-like cortical plasticity in Alzheimer’s disease patients. J Alzheimers Dis. 2012;31(3):593-599. doi:10.3233/JAD-2012-120532 22647254

[42] Choung JS, Kim JM, Ko M-H, Cho DS, Kim M. Therapeutic efficacy of repetitive transcranial magnetic stimulation in an animal model of Alzheimer’s disease. Sci Rep. 2021;11(1):437. doi:10.1038/s41598-020-80147-x 33432077 PMC7801521

[43] Shang Y, Wang X, Shang X, . Repetitive transcranial magnetic stimulation effectively facilitates spatial cognition and synaptic plasticity associated with increasing the levels of BDNF and synaptic proteins in Wistar rats. Neurobiol Learn Mem. 2016;134:369-378. doi:10.1016/j.nlm.2016.08.016 27555233

[44] Cheeran B, Talelli P, Mori F, . A common polymorphism in the brain-derived neurotrophic factor gene (BDNF) modulates human cortical plasticity and the response to rTMS. J Physiol. 2008;586(23):5717-5725. doi:10.1113/jphysiol.2008.159905 18845611 PMC2655403

[45] Huang Z, Tan T, Du Y, . Low-frequency repetitive transcranial magnetic stimulation ameliorates cognitive function and synaptic plasticity in APP23/PS45 mouse model of Alzheimer’s disease. Front Aging Neurosci. 2017;9:292. doi:10.3389/fnagi.2017.00292 28955219 PMC5600921

[46] Freedberg M, Reeves JA, Toader AC, Hermiller MS, Voss JL, Wassermann EM. Persistent enhancement of hippocampal network connectivity by parietal rTMS is reproducible. eNeuro. 2019;6(5):ENEURO.0129-19.2019. doi:10.1523/ENEURO.0129-19.2019 31591137 PMC6795558

[47] Wagner AD, Shannon BJ, Kahn I, Buckner RL. Parietal lobe contributions to episodic memory retrieval. Trends Cogn Sci. 2005;9(9):445-453. doi:10.1016/j.tics.2005.07.001 16054861

[48] Velioglu HA, Hanoglu L, Bayraktaroglu Z, . Left lateral parietal rTMS improves cognition and modulates resting brain connectivity in patients with Alzheimer’s disease: possible role of BDNF and oxidative stress. Neurobiol Learn Mem. 2021;180:107410. doi:10.1016/j.nlm.2021.107410 33610772

[49] Jia Y, Xu L, Yang K, . Precision repetitive transcranial magnetic stimulation over the left parietal cortex improves memory in Alzheimer’s disease: a randomized, double-blind, sham-controlled study. Front Aging Neurosci. 2021;13:693611. doi:10.3389/fnagi.2021.693611 34267648 PMC8276073

[50] Drumond Marra HL, Myczkowski ML, Maia Memória C, . Transcranial magnetic stimulation to address mild cognitive impairment in the elderly: a randomized controlled study. Behav Neurol. 2015;2015:287843. doi:10.1155/2015/287843 26160997 PMC4487699

[51] Cole EJ, Stimpson KH, Bentzley BS, . Stanford accelerated intelligent neuromodulation therapy for treatment-resistant depression. Am J Psychiatry. 2020;177(8):716-726. doi:10.1176/appi.ajp.2019.19070720 32252538

