# Error in Author Affiliations

**DOI:** 10.1001/jamanetworkopen.2024.26187

**Published:** 2024-07-22

In the Original Investigation titled “Effectiveness of Personalized Hippocampal Network–Targeted Stimulation in Alzheimer Disease: A Randomized Clinical Trial,”^1^ published May 6, 2024, an institutional affiliation was omitted. Dr S. Kim is affiliated with the Department of Healthcare Digital Engineering, Hanyang University. This article has been corrected.^1^
