# Supplementary material for: Effectiveness of Personalized Hippocampal Network–Targeted Stimulation in Alzheimer Disease: A Randomized Clinical Trial
Source: JAMA Netw Open. 2024 May 6;7(5):e249220. doi: 10.1001/jamanetworkopen.2024.9220 (PMC11074813; doi:10.1001/jamanetworkopen.2024.9220)
Supplement: Supplement 1. — eAppendix 1. Eligibility Criteria eAppendix 2. Randomization and Masking eAppendix 3. Brain MRI Acquisition eAppendix 4. MRI Data Preprocessing eAppendix 5. Aβ PET Acquisition and Definition of Aβ Positivity eAppendix 6. CSF Collection and AD Biomarker Analysis eAppendix 7. Stimulation Procedure With Target Selection Based on fMRI Data Analysis eAppendix 8. fMRI Data Analysis eAppendix 9. Sample Size eTable 1. Characteristics of Study Participants Who Completed the Trial eTable 2. Paired Comparison of the Neuropsychological Profiles of Each Visit Across the Groups eTable 3. Original and MNI Coordinates for Individual Patients eFigure. Modeling for a Personalized 3D-Printed Frame as a TMS Guide eAppendix 10. Effect of the Hippocampal Network–Targeted rTMS on ADAS-Cog Change According to the Diagnosis eReferences [file jamanetwopen-e249220-s001.pdf]

Supplemental Online Content

Jung YH, Jang H, Park S, et al. Effectiveness of personalized hippocampal network-targeted stimulation in Alzheimer disease. *JAMA Netw Open.* 2024;7(5):e249220. doi:10.1001/jamanetworkopen.2024.9220

eAppendix 1. Eligibility Criteria

eAppendix 2. Randomization and Masking

eAppendix 3. Brain MRI Acquisition

eAppendix 4. MRI Data Preprocessing

eAppendix 5. Aβ PET Acquisition and Definition of Aβ Positivity

eAppendix 6. CSF Collection and AD Biomarker Analysis

eAppendix 7. Stimulation Procedure With Target Selection Based on fMRI Data Analysis

eAppendix 8. fMRI Data Analysis

eAppendix 9. Sample Size

eTable 1. Characteristics of Study Participants Who Completed the Trial

eTable 2. Paired Comparison of the Neuropsychological Profiles of Each Visit Across the Groups

eTable 3. Original and MNI Coordinates for Individual Patients

eFigure 1. Flow diagram From Screening to the Follow-Up of Study Participants

eFigure 2. Modeling for a Personalized 3D-Printed Frame as a TMS Guide

eAppendix 10. Effect of the Hippocampal Network-Targeted rTMS on ADAS-Cog Change According to the Diagnosis

eReferences

This supplemental material has been provided by the authors to give readers additional information about their work.

© 2024 Jung YH et al. *JAMA Network Open.*

eAppendix 1. Eligibility criteria

Detailed inclusion criteria was as follow: 1) aged between 55 and 90 years; 2) diagnosed as either mild cognitive impairment due to AD or mild AD dementia based on National Institute on Aging–Alzheimer's Association criteria,[1] with amyloid positivity determined by PET or CSF testing; 3) Objective memory impairment as indicated by at least two standard deviations below the age- and education-adjusted mean in verbal language test (SVLT) or Rey-copy figure test (RCFT) delayed recall tests; 4) ≥ 18 of Mini-Mental State Examination (MMSE) score; 5) no history of epilepsy; 6) normal in electroencephalography (EEG): 7) no myocardial infarcts or arrhythmia in electrocardiography (ECG); 8) those who are literate; 9) those who voluntarily submitted written consents.

We excluded participants 1) with other medical or surgical diseases that caused dementia; 2) with severe white matter hyperintensities (WMH) on magnetic resonance image (MRI), which were defined as deep WMH ≥25 mm and periventricular WMH ≥10 mm; 3) who could not undergo MRI due to side effects related to contrast agents, or claustrophobia; 4) who have implanted medical devices that are susceptible to electronic disturbance, such as pacemaker, or detachable metallic materials (prosthetics, braces, cochlear implants etc.); 5) who have undergone cerebrovascular surgery, such as coiling clipping, and carotid artery stenting; 6) who have difficulty breathing when sitting; 7) who have lost consciousness for more than one hour due to causes other than general anesthesia; 8) who have been hospitalized with a head trauma; 9) who cannot read even with glasses due to reduced vision; 10) who have difficulty understanding conversations due to hearing impairment even with a hearing aid; 11) who have taken ototoxic drugs or have been exposed to loud noises.[2]

© 2024 Jung YH et al. *JAMA Network Open.*

eAppendix 2. Randomization and Masking

Using six-block randomization, the participants were randomly allocated to the rTMS and sham groups. Randomization was performed and assigned independently by an external statistician. Dedicated technicians performed stimulation. An expert neurologist who enrolled participants, expert neuropsychologists who performed cognitive evaluations, and participants/their caregivers were all blinded to the treatment allocation.

© 2024 Jung YH et al. *JAMA Network Open.*

eAppendix 3. Brain MRI acquisition

An Achieva 3.0 Tesla MRI scanner (Philips, Best, The Netherlands) was used to acquire 3D T1 turbo field echo (TFE) MRI data from all patients using the following imaging parameters: sagittal slice thickness, 1.0 mm; over contiguous slices with 50 % overlap; no gap; repetition time, 9.9 ms; echo time, 4.6 ms; flip angle, 8 degrees; matrix size of 365 × 512 × 512 voxels reconstructed from 240 phase encoding steps over a field of view, 182 mm × 240 mm × 240 mm. Resting fMRI was performed using an echo planar imaging (EPI) sequence with the following parameters: 300 volumes with a repetition time of 2000 ms, axial slice thickness of 2 mm, no gap, echo time of 35 ms, flip angle of 90 °, and matrix size of 128 × 128 × 60 voxels over a field of view of 218 mm × 218 mm.

© 2024 Jung YH et al. *JAMA Network Open.*

eAppendix 4. MRI data preprocessing

Anatomical and resting-state fMRI were preprocessed using the "afni_proc.py" script from the AFNI (Analysis of Functional Neuroimages)[4], with filtering out volumes that exceeded thresholds of 0.4 mm Euclidean motion per TR (option, "-regress_censor_motion") and 5% of voxels marked as outliers (option, "-regressor_censor_outliers option") during the censoring and despiking stages. The preprocessing included slice-timing correction, motion correction, and registering to skull-stripped anatomical data, which were non-linearly transformed into the Montreal Neurological Institute (MNI) template. The original fMRI data were resampled to a 1.5 x 1.5 x 1.5 mm resolution. Gaussian smoothing was applied to all images using a 4 × 4 × 4-mm full-width at the half-maximum kernel, and the time series were scaled to have a mean of 100 and a range between 0 and 200. For connectivity analysis, we regressed out signals from the white matter and ventricle regions, which were defined using FreeSurfer program[5] "fs_ap_latvent" and "fs_ap_wm", respectively, as well as a global signal calculated from averaging signals from a whole brain mask. We also regressed out 12 signals of non-interest, including six head-motion realignment parameters and their derivatives, as well as three signals modeling up to second-order polynomial trends in the fMRI data.

© 2024 Jung YH et al. *JAMA Network Open.*

eAppendix 5. Aβ PET acquisition and definition of Aβ positivity

Among 44 patients, 42 patients underwent Aβ PET (florbetaben, N = 15; flutemetamol, N = 27). For 18F-florbetaben PET, a 20-minute emission PET scan in dynamic mode (consisting of 4 × 5 min frames) was performed 90 min after bolus injection of a mean dose of 381 MBq 18F-florbetaben into an antecubital vein. For flutemetamol PET, a 20-minute emission static PET scan in dynamic mode (consisting of 4 x 5 min frames) was performed 90 min after bolus injection of a mean dose of 185 MBq flutemetamol into an antecubital vein.

We defined Aβ positivity (Aβ+) for the two different types of PET images as follows: (1) visual rating score on florbetaben PET of 2 or 3 according to the brain Aβ plaque load scoring system[6] and (2) positive visual interpretation of flutemetamol PET in any one of the five brain regions (frontal, parietal, posterior cingulate and precuneus, striatum, and lateral temporal lobes) in either hemisphere.[7]

© 2024 Jung YH et al. *JAMA Network Open.*

eAppendix 6. CSF collection and AD biomarker analysis

CSF samples were collected from a lumbar puncture performed in the L3-4 or L4-5 intervertebral spaces using a 20 or 22G needle for the two patients. CSF samples were collected in 15-mL polypropylene tubes at the time of the tap and immediately sent to the Samsung Medical Center laboratory. After samples were centrifuged at 2000 g for 10 min within 4 hours after collection, aliquots (1 mL) prepared from these samples were immediately stored in bar-code-labeled polypropylene vials at −70 °C. In our laboratory, we ran assays for CSF biomarkers - Amyloid-β $_{(1–42)}$ (Aβ42), total tau, and 181 phosphorylated tau - using INNOTEST enzyme-linked immunosorbent assay (ELISA) kits (Fujirebio Europe N.V.). Amyloid positivity was defined as a CSF Aβ42 value lower than 667.9.[8]

© 2024 Jung YH et al. *JAMA Network Open.*

eAppendix 7. Stimulation procedure with target selection based on fMRI data analysis

Resting motor threshold was determined for individual patients by placing a magnetic coil over the motor cortex and adjusting the stimulation intensity to elicit a visible contraction of the patient's hand. The intensity was adjusted to 100 % of the motor threshold in the left parietal area and was treated during the same daily session (five days per week). Each area was stimulated with 40 trains (20 Hz for 2 s at 40 pulses/train), equating to 1600 pulses applied daily. All rTMS applications followed the guidelines for the therapeutic use of rTMS.[9] The sham group was exposed to recorded pulse sounds without magnetic stimulation.

We identified individualized target locations of rTMS from connectivity analysis using 10 min resting-state fMRI data acquired during the pretreatment session, following a procedure in previous studies.[10-12] We first defined a hippocampal seed in the body of the left hippocampus around the MNI (-29, -25, -13). Then, the location was used for seed-based connectivity (Pearson correlation) analysis using an "InstaCorr" function of the AFNI software (Version 23.1.01)[4] with a 3-mm radius spherical mask, six motion-related regressors of non-interest and a bandpass filtering between 0.01 and 0.1 Hz. We searched for a simulation target within the parietal cortex posterior to the postcentral sulcus, including superior and inferior parietal cortices. The left lateral parietal location demonstrating the highest connectivity with the hippocampal target was selected as the stimulation target.[10,12] For one patient from the rTMS group, we used a default stimulation location, MNI (-48, -70, 38), because no robust connectivity was found in the left lateral parietal region. The original and MNI coordinates for individual patients are summarized in **Table 1**. Most targets were found in the inferior parietal cortex, and few were found in the superior parietal cortex. Finally, we produced a personalized 3-D printed frame with space for an rTMS coil, the center of which was aligned with the original target coordinate (Anymedi Inc., Korea). The frame was modeled such that the coil handle was approximately perpendicular to the local gyrus in the parietal region, equivalently pointing posteriorly at a 45-degree angle relative to the medial-sagittal plane. Throughout the study, the target site for each patient remained consistent because of the use of a custom 3D-printed frame. This frame was used to secure the TMS coil in place at the precise stimulation site, ensuring accurate and consistent targeting of the desired brain region. rTMS sham stimulation was applied using a sham coil positioned in the corresponding target area. There were technical issues in producing a personalized 3-D printed frame for several patients recruited earlier (eight in the rTMS group and nine in the sham group). Consequently, the target location guided by the frame deviates slightly from the optimal location determined by the target identification procedure described previously, but the deviations were negligible in practice.

© 2024 Jung YH et al. *JAMA Network Open.*

eAppendix 8. fMRI data analysis

Using the preprocessed fMRI data described earlier, we conducted connectivity analysis utilizing a hippocampal seed at the MNI (-29, -25, -13), a reference left hippocampal body coordinate used in a previous rTMS study.[11] For individual patients, we first calculated whole-brain seed-based connectivity (Pearson correlation) for pre- and post-treatment fMRI data and their differences. We then performed group-level voxel-wise t-tests using AFNI's 3dttest++ function between the rTMS and sham groups to identify the rTMS-responsive region showing more significant changes in connectivity with the hippocampus than the sham group. Building on previous studies that employed a similar hippocampal directly targeted stimulation (HITS) protocol,[10-14] we defined the precuneus as the region of interest (ROI), where we hypothesized an enhancement in connectivity with the hippocampus due to rTMS. The precuneus ROI was obtained from Neurosynth, a large-scale meta-analytic fMRI database[15] (https://neurosynth.org, accessed on July 2nd, 2023), using the term **"**precuneus,**"** which retrieved 1,014 studies and 36,712 activations. We adjusted the threshold z-score to seven, including 3808 contiguous voxels (12,852 mm^3). We used a relatively lenient voxel-wise threshold of $P < 0.05$, owing to the limited sample size, as in a previous study.[10] A Monte Carlo simulation (AFNI 3dttest++ function with a -Clustsim option) was used to determine the spatial extent threshold of 195 contiguous supra-threshold voxels (658 mm^3) within the precuneus ROI to provide a cluster-wise corrected threshold of $P < 0.05$. Despite the lenient voxel-wise threshold of $P < 0.05$, the small-volume correction with a predefined ROI would compromise the risk of false positives while increasing the sensitivity of identifying true positives.

The identified significant rTMS-responsive region within the precuneus ROI was used for subsequent correlation analysis between hippocampal-cortical fMRI connectivity and ADAS-Cog scores, the primary clinical outcomes. Based on previous studies using rTMS to improve associative memory[10,13], we hypothesized that increased interaction between the hippocampus and posterior-medial cortical regions significantly correlates with improvements in cognitive performance. To test this hypothesis, we calculated changes in hippocampal-cortical resting-state functional connectivity and cognitive performance from baseline to post-treatment (immediately after 4-week treatment, V1) in both the rTMS and sham groups. Among the various cognitive scores tested, we focused on the primary outcome, ADAS-Cog, which was found to be significantly improved immediately after the fourth week of rTMS treatment (V1) as well as the fourth week of follow-up (V2) compared with the sham group (Figure 4). Due to the limited sample size of the fMRI data, we used the Spearman rank correlation instead of the Pearson correlation. We used AFNI (Version 23.1.01), Nilearn Python packages for the fMRI data analyses, and a custom Python script (Version 3.9.16) to visualize the whole-brain voxel-wise analysis and correlation analysis results.

© 2024 Jung YH et al. *JAMA Network Open.*

eAppendix 9. Sample size

The sample size was estimated based on a previous study[3]. The changes of ADAS-Cog scores from baseline to the six weeks were 3.76 (standard error [SE] 1.32, N=7) and 0.47 (SE 1.18, N=8) in the rTMS and sham groups, respectively. With the calculated standard deviation in changes of 3.41, 17 patients per group achieved 80 % power with a significance level of $P < 0.05$ using a two-sided, two-sample, unequal-variance t-test. Since we assumed a dropout rate of about 15%, the final sample size was estimated 20 participants per group.

© 2024 Jung YH et al. *JAMA Network Open.*

eTable 1. Characteristics of Study Participants Who Completed the Trial

**Characteristic**	**Participants, No. (%)**	
	**Sham (n = 12)**	**rTMS (n = 18)**
Age, mean (SD), y	69.9 (7.1)	69.8 (9.1)
Sex		
Female	6 (50)	12 (67)
Male	6 (50)	6 (33)
Education year, median (IQR)	16 (12.0-17.5)	16 (12.8-16)
APOE ε4 carrier	10 (83)	12 (71)[a]
Vascular risk factors		
Diabetes	2 (17)	3 (17)
Hypertension	2 (25)	6 (33)
Hyperlipidemia	6 (50)	8 (44)
MMSE score, mean (SD)	22.5 (3.58)	23.9 (3.52)
CDR-SOB, median (IQR)	4.5 (1.8-6.6)	3.5 (2.3-5.1)
Medication use		
Acetylcholine esterase inhibitor	12 (100)	18 (100)
Memantine	2 (17)	3 (17)

Abbreviations: APOE, apolipoprotein E; CDR-SOB, clinical dementia rating-sum of boxes; MMSE, Mini-Mental Status Examination; rTMS, repetitive transcranial magnetic stimulation.

[a]Data missing for 1 participant.

© 2024 Jung YH et al. *JAMA Network Open.*

eTable 2. Paired comparison of the neuropsychological profiles of each visit across the groups

Neuro -psycho logical test	Sham				rTMS			
	V1- V0		V2- V0		V1- V0		V2- V0	
	coeff (SE)	p-value	coeff (SE)	p-value	coeff (SE)	p-value	coeff (SE)	p-value
**ADAS-Cog, total**	1.2 (1.3)	0.94	1.6 (1.3)	0.79	-3.2 (1.0)	0.014*	-3.5 (1.0)	0.01*
**Memory**	-0.8 (1.1)	0.98	-0.6 (1.1)	0.99	-2.4 (0.9)	0.11	-3.5 (0.9)	0.005
**Language**	-0.3 (0.6)	0.56	-0.9 (0.6)	0.99	0.1 (0.5)	1.0	0.7 (0.5)	0.69
**Praxis**	-0.5 (0.5)	0.93	0.2 (0.5)	1.0	0.3 (0.4)	0.98	0.2 (0.4)	1.00
**MMSE**	0 (0.6)	1.0	0.08 (0.6)	1.0	0.6 (0.5)	0.8	0.6 (0.5)	0.84
**MOCA**	0.8 (0.9)	0.96	-0.5 (0.9)	1.0	0.5 (0.7)	0.98	-0.2 (0.8)	1.00
**CDR-SOB**	0.4 (0.2)	0.40	0.6 (0.2)	0.11	-0.2 (0.2)	0.96	-0.2 (0.2)	0.88
**SIADL**	1.0 (0.6)	0.53	1.5 (0.6)	0.11	-0.1 (0.5)	1.0	-1,0 (0.5)	0.49
**Frontal-executive tests**								
**COWAT-semantic**	0.5 (1.0)	0.99	-0.2 (1.0)	1.0	-0.2 (0.7)	1.0	-1.0 (0.8)	0.80
**COWAT-phonemic**	-0.4 (1.7)	1.00	-0.4 (1.7)	1.0	0.3 (1.3)	1.0	1.9 (1.4)	0.76
**K-TMT-B**	-4.8 (12.2)	1.00	-17.8 (12.2)	0.69	-1.0 (9.3)	1.0	4.1 (9.8)	1.0
**Stroop color**	7.6 (3.8)	0.35	2.7 (3.8)	0.98	-1.4 (2.9)	1.0	-4.5 (3.1)	0.70
**CANTAB**								
**DMS**	-5.4 (4.9)	0.87	-0.4 (5.0)	1.0	0.4 (3.9)	1.00	2.7 (4.1)	0.99
**PAL**	1.9 (2.2)	0.95	1.7 (2.3)	0.98	-2.5 (1.8)	0.71	-3.9 (1.9)	0.32
**PRM**	7.7 (5.8)	0.77	13.0 (6.0)	0.19	1.8 (4.6)	1.0	3.6 (4.9)	0.98
**RTI**	9.7 (8.3)	0.85	8.8 (8.5)	0.91	13.5 (6.6)	0.34	2.6 (7.1)	1.0
**RVP**	-0.01 (0.02)	0.98	0.01 (0.02)	1.0	0 (0.02)	1.0	0.01 (0.02)	1.0
**SWMS**	0.5 (0.4)	0.89	0.9 (0.4)	0.35	0 (0.3)	1.0	-0.02 (0.4)	1.0

© 2024 Jung YH et al. *JAMA Network Open.*

eTable 3. Original and MNI coordinates for individual patients

	Original Coordinate			MNI Coordinate		
Patient ID*	X	Y	Z	X	Y	Z
**Sham group (N=12)**						
S05	52.62	43.09	38.13	-52	-52	37
S06	63.35	35.19	3.38	-58	-66	26
S10	51.68	36.98	21.76	-47	-40	63
S14	36.48	53.38	37.22	-33	-77	34
S17	47.86	45.73	28.48	-53	-58	26
S18	39.59	53.14	96.31	-38	-57	63
S20	54.14	51.53	32.51	-42	-78	32
S24	48.39	55.23	34.11	-60	-68	34
S25	37.79	52.76	48.30	-52	-59	34
S27	53.71	51.31	7.11	-56	-65	33
S32	60.31	43.37	43.24	-58	-64	27
S36	47.29	57.04	-4.09	-42	-72	34
**rTMS group (N=18)**						
S09	44.25	45.32	16.96	-54	-65	11
S11	57.12	36.04	36.86	-62	-49	35
S12	44.39	51.18	37.45	-42	-60	29
S15	50.47	49.97	3.29	-49	-76	27
S19	37.44	56.55	55.91	-26	-71	43
S21	49.20	66.83	54.44	-52	-78	32
S22	44.80	68.90	44.53	-47	-79	50
S26	53.13	54.00	29.68	-52	-73	35
S31	59.31	52.96	20.00	-56	-65	24
S33	47.31	44.23	70.38	-55	-49	30
S37	47.77	58.05	-11.92	-51	-73	26
S38	55.20	48.76	1.81	-58	-52	30
S40	43.82	67.16	1.95	-48	-70	38
S43	53.77	36.74	51.25	-59	-46	38
S46	37.96	43.59	25.30	-46	-57	25
S47	50.50	61.44	12.48	-52	-72	38
S48	51.48	60.49	19.43	-47	-81	32
S50	-47.38	-49.67	55.59	-47	-59	39

*Patient ID was given in the order of registration

© 2024 Jung YH et al. *JAMA Network Open.*

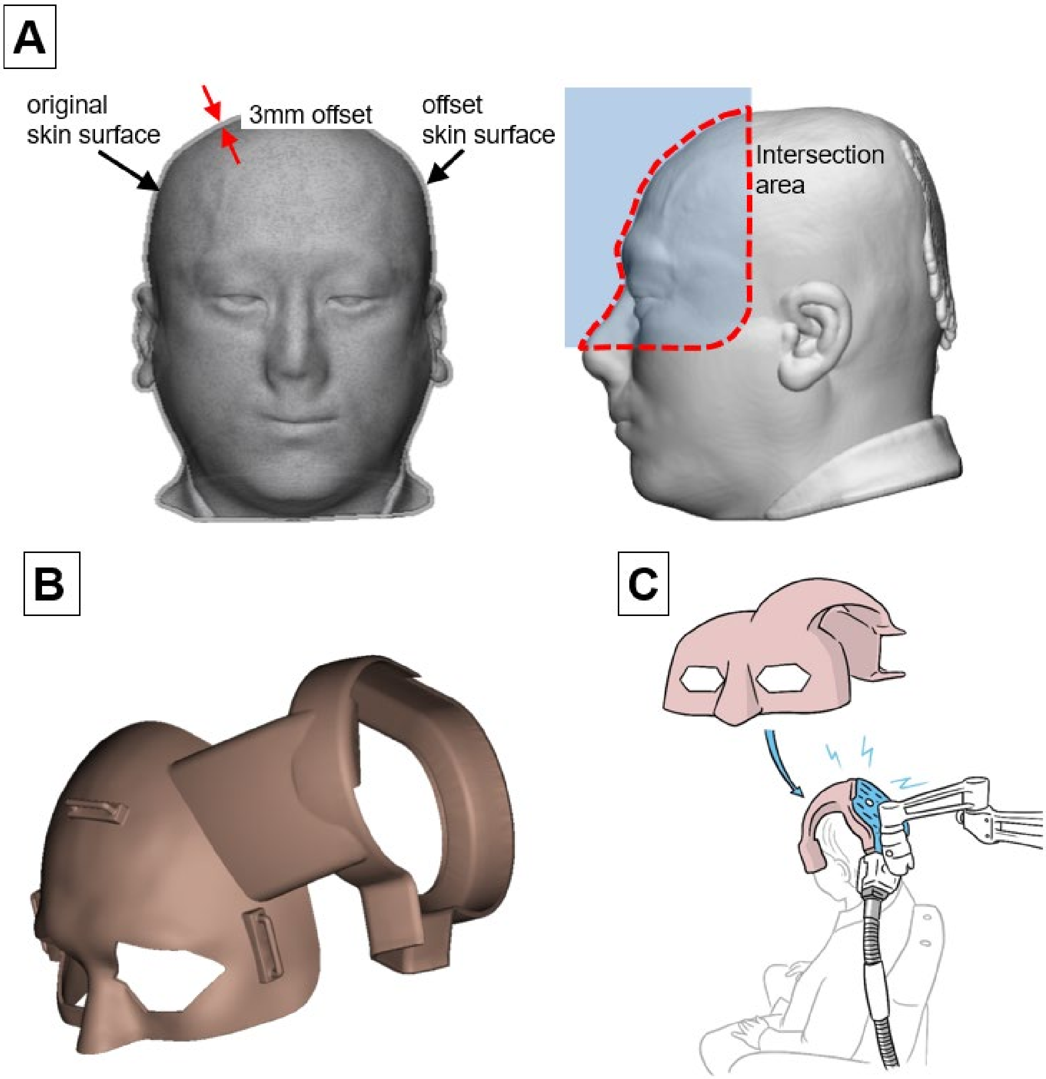

eFigure. Modeling for a personalized 3D-printed frame as a TMS guide. (A) Personalized 3D-printed frame (B) Target point on a structural MRI and 3D rendering of TMS module mounting on an individual participant (C) Actual clinical trials with a TMS coil inserted in a 3D-printed frame. TMS: transcranial magnetic stimulation, MRI: magnetic resonance image

© 2024 Jung YH et al. *JAMA Network Open.*

eAppendix 10. Effect of the hippocampal network-targeted rTMS on ADAS-Cog change according to the diagnosis

When we replicated the main analyses to assess the impact of rTMS treatment on ADAS-Cog changes within aMCI and mild ADD groups separately, a significant interaction was observed at V2 (Coefficient (SE) -4.5(1.6), P = 0.007) and V1 (Coef (SE) -4.2(1.6), P=0.013) in the aMCI subgroup. On the other hand, in the ADD subgroup, the interaction demonstrated a trend toward significance at V2 (Coef (SE) -5.8 (2.8), P=0.05) and V1 (Coef (SE)-4.3 (2.8), P=0.13). When we extended the analysis to the entire group, incorporating the interaction term of intervention * visit * diagnosis (aMCI vs. ADD), the interaction was not significant both at V1 (P for interaction = 0.900) and at V2 (P for interaction = 0.653), indicating no differences in intervention effects based on cognitive status.

Table. Effect of the hippocampal network-targeted rTMS on ADAS-Cog change according to the diagnosis

Diagnosis	group	ADAS-cog score			Intervention effect (group * visit)			
		V0 (baseline)	V1 (4wk)	V2 (8wk)	4 week		8 week	
					Coef (SE)	P value	Coef(SE)	P value
aMCI (n=16)	Sham (n=6)	29.0±5.8	29.3±3.8	28.7±5.4	-4.5 (1.6)	0.007	-4.2 (1.6)	0.013
	rTMS (n=10)	27.1±8.71	23.1±8.2	22.7±9.0				
ADD (n=14)	Sham (n=6)	34.3±7.7	36.2±8.5	38.0±7.2	-4.3 (2.8)	0.13	-5.8 (2.8)	0.05
	rTMS (n=8)	35.1±9.3	33.4±12.7	33.1±11.4				

© 2024 Jung YH et al. *JAMA Network Open.*

eReferences

1. McKhann GM, Knopman DS, Chertkow H, et al. The diagnosis of dementia due to Alzheimer's disease: Recommendations from the National Institute on Aging-Alzheimer's Association workgroups on diagnostic guidelines for Alzheimer's disease. 2011;7(3):263-269.
2. Rossi S, Antal A, Bestmann S, et al. Safety and recommendations for TMS use in healthy subjects and patient populations, with updates on training, ethical and regulatory issues: Expert Guidelines. 2021;132(1):269-306.
3. Rabey JM, Dobronevsky E, Aichenbaum S, Gonen O, Marton RG, Khaigrekht M. Repetitive transcranial magnetic stimulation combined with cognitive training is a safe and effective modality for the treatment of Alzheimer's disease: a randomized, double-blind study. *Journal of neural transmission (Vienna, Austria : 1996)*. May 2013;120(5):813-9. doi:10.1007/s00702-012-0902-z
4. Cox RW. AFNI: software for analysis and visualization of functional magnetic resonance neuroimages. *Comput Biomed Res*. Jun 1996;29(3):162-73. doi:10.1006/cbmr.1996.0014
5. Fischl B. FreeSurfer. *Neuroimage*. Aug 15 2012;62(2):774-81. doi:10.1016/j.neuroimage.2012.01.021
6. Barthel H, Gertz H-J, Dresel S, et al. Cerebral amyloid-β PET with florbetaben (18F) in patients with Alzheimer's disease and healthy controls: a multicentre phase 2 diagnostic study. 2011;10(5):424-435.
7. Farrar G, Molinuevo JL, Zanette MJEJoNM, Imaging M. Is there a difference in regional read [18 F] flutemetamol amyloid patterns between end-of-life subjects and those with amnestic mild cognitive impairment? 2019;46:1299-1308.
8. Lee J, Jang H, Kang SH, et al. Cerebrospinal fluid biomarkers for the diagnosis and classification of Alzheimer's disease spectrum. 2020;35(44)
9. Lefaucheur J-P, André-Obadia N, Antal A, et al. Evidence-based guidelines on the therapeutic use of repetitive transcranial magnetic stimulation (rTMS). 2014;125(11):2150-2206.
10. Wang JX, Rogers LM, Gross EZ, et al. Targeted enhancement of cortical-hippocampal brain networks and associative memory. 2014;345(6200):1054-1057.
11. Kim S, Nilakantan AS, Hermiller MS, Palumbo RT, VanHaerents S, Voss JL. Selective and coherent activity increases due to stimulation indicate functional distinctions between episodic memory networks. *Sci Adv*. Aug 2018;4(8):eaar2768. doi:10.1126/sciadv.aar2768
12. Nilakantan AS, Mesulam M-M, Weintraub S, Karp EL, VanHaerents S, Voss JLJN. Network-targeted stimulation engages neurobehavioral hallmarks of age-related memory decline. 2019;92(20):e2349-e2354.
13. Warren KN, Hermiller MS, Nilakantan AS, Voss JLJE. Stimulating the hippocampal posterior-medial network enhances task-dependent connectivity and memory. 2019;8:e49458.

© 2024 Jung YH et al. *JAMA Network Open.*

14. Freedberg M, Cunningham CA, Fioriti CM, et al. Multiple parietal pathways are associated with rTMS-induced hippocampal network enhancement and episodic memory changes. 2021;237:118199.

15. Yarkoni T, Poldrack RA, Nichols TE, Van Essen DC, Wager TD. Large-scale automated synthesis of human functional neuroimaging data. *Nat Methods*. Jun 26 2011;8(8):665-70. doi:10.1038/nmeth.1635

© 2024 Jung YH et al. *JAMA Network Open.*
