# Supplementary material for: Effectiveness of Personalized Hippocampal Network–Targeted Stimulation in Alzheimer Disease: A Randomized Clinical Trial
Source: JAMA Netw Open. 2024 May 6;7(5):e249220. doi: 10.1001/jamanetworkopen.2024.9220 (PMC11074813; doi:10.1001/jamanetworkopen.2024.9220)
Supplement: Supplement 2. — Trial Protocol [file jamanetwopen-e249220-s002.pdf]

Clinical Trial Protocol

The effects of transcranial magnetic stimulation (ALTMS) on cognition in the patients with Alzheimer's disease: A single centered, randomized, evaluator-blinded, exploratory clinical trial

Protocol No. : TMS-Alzheimer, Version No. 4.4

Principal investigator: Hyemin Jang

CONTENTS

Protocol No. : TMS-Alzheimer, Version No. 4.4

<Summary of Clinical Trial Protocol> -- 3-7

1. Clinical Trial Title -- 8

2. Clinical trial Institution --- 9

3. Principal Investigator, collaborator, and Coordinator -------------------------------- 10

4. Medical Device Manager -- 11

5. Clinical Trial Sponsor -- 12

6. Objective and Backgrounds -- 13-15

7. Inclusion Criteria, Exclusion Criteria, and Sample Size Determination -----------16-18

8. Clinical Trial Duration --- 19

9. Methods -- 20-24

10. Observation Items and Observation Methods ---------------------------------- 25-35

11. Anticipated Adverse Effects and Usage Precautions --------------------------- 36-37

12. Termination, Dropout Criteria, and Re-screening ------------------------------ 38-40

13. Efficacy Evaluation Criteria, Assessment Methods, and Interpretation -------- 41-42

14. Safety Evaluation Criteria, Assessment Methods & Adverse Event Reporting 43-44

15. Reference --- 45

< Clinical Trial Protocol>

<table>
<tr><td>Objectives</td><td colspan="3">This clinical trial aims to demonstrate the improvement of memory and cognitive function using transcranial magnetic stimulation applied to the patients with Alzheimer's disease.</td></tr>
<tr><td>Clinical Trial Institution</td><td colspan="3">Samsung Medical Center</td></tr>
<tr><td>Clinical Trial Sponsor</td><td colspan="3">Hyemin Jang, Professor, Samsung Medical Center</td></tr>
<tr><td>Clinical trial duration</td><td colspan="3">2018.10~2020.10 (24 months)</td></tr>
<tr><td>Clinical trial design</td><td>☐ non-randomized allocation
■ randomized allocation</td><td>■ single arm design
■ parallel desing
☐ cross-over design</td><td>☐ open labeled
☐ single blinded
☐ double blinded
■ evaluator blinded</td></tr>
<tr><td rowspan="2">Subject</td><td>Inclusion criteria</td><td colspan="2">1) aged between 55 and 90 years
2) diagnosed as either mild cognitive impairment due to AD or mild AD dementia on the basis of National Institute on Aging–Alzheimer’s Association criteria, with amyloid positivity determined by PET or CSF testing
3) Objective memory impairment as indicated by at least 2 standard deviations below the age- and education-adjusted mean in verbal language test (SVLT) or Rey-copy figure test (RCFT) delayed recall tests
4) ≥ 18 of Mini-Mental State Examination (MMSE) score
5) no history of epilepsy
6) normal in electroencephalography (EEG)
7) no myocardial infarcts or arrhythmia in electrocardiography (ECG)
8) those who are literate
9) those who voluntarily submitted written consents.</td></tr>
<tr><td>Exclusion criteria</td><td colspan="2">1) with other medical or surgical diseases that caused</td></tr>
</table>

		dementia;<br>2) with severe white matter hyperintensities (WMH) on magnetic resonance image (MRI), which were defined as deep WMH ≥25 mm and periventricular WMH ≥10 mm;<br>3) who could not undergo MRI due to side effects related to contrast agents, or claustrophobia; 4) who have implanted medical devices that are susceptible to electronic disturbance, such as pacemaker, or detachable metallic materials (prosthetics, braces, cochlear implants etc.);<br>5) who have undergone cerebrovascular surgery, such as coiling clipping, and carotid artery stenting;<br>6) who have difficulty breathing when sitting;<br>7) who have lost consciousness for more than one hour due to causes other than general anesthesia;<br>8) who have been hospitalized with a head trauma;<br>9) who cannot read even with glasses due to reduced vision;<br>10) who have difficulty understanding conversations due to hearing impairment even with a hearing aid;<br>11) who have taken ototoxic drugs or have been exposed to loud noises.	
	Number of subjects	rTMS group	16 participants
		Sham group	16 participants
Methods	Target selection	Using the findings from resting-state functional MRI analysis, a specific area in the left lateral parietal region, characterized by the strongest connectivity with the hippocampus, was pinpointed. This particular area was chosen as the focal point for stimulation and precisely positioned using a medical guide during the rTMS treatment.	

	Stimulation procedure	Intensity : motor threshold 100%<br>The motor threshold was determined for individual patients by placing a magnetic coil over the motor cortex and adjusting the stimulation intensity to elicit a visible contraction of the patient's hand. The intensity was adjusted to 100 % of the motor in the left parietal area and was treated during the same daily session.<br>Number of pulses : 1600 pulses<br>Each area was stimulated with 40 trains (20 Hz for 2 s at 40 pulses/train), equating to 1600 pulses applied daily. All rTMS applications followed the guidelines for the therapeutic use of rTMS. The sham group was exposed to recorded pulse sounds without magnetic stimulation.<br>Duration:<br>- duration: four weeks<br>The stimulation takes approximately 20 minutes per session, and it is administered five times a week (Monday to Friday) for a total of 20 sessions over the course of four weeks.
	Medical device	1. ALTMS<br>- manufacturer: ㈜ REMED<br>- magnetic field intensity: 2.5 T<br>- magnetic field frequency: max 100Hz, min 0.1Hz<br>- stimulation time: user-adjustable in increments of 0.1 seconds<br>- output waveform: Symmetric biphasic pulse<br>- pulse width: 350 μs<br>- magnetic field generation transducer: 8-coil type<br>- weight: 60kg<br>- power recruitment: 200~ 240 Vac 50/60 Hz<br>2. stimulator positioning guide<br>- manufacturer: Anymed corp<br>-3D-printed medical guide for accurate positioning of the transducer of the medical electromagnetic generator on the patient's skull to provide precise guidance for treatment location information.
	rTMS group	rTMS stimulation

	Sham group	Sham coil stimulation	
outcomes	Outcome measures	Primary outcome	Change in ADAS-Cog scores before and 8 weeks after stimulation in the rTMS group.
		Secondary outcome	<Comparison Before and After rTMS Stimulation><br>The change in scores at baseline, 4 weeks, and 8 weeks based on neuropsychological assessments (K-MMSE, K-MoCA, CDR-SOB, ADAS-Cog/COWAT, Stroop, TMT, RVP, SWM, PRM) in the rTMS stimulation group.<br>The change in total scores derived from K-GDS short form between baseline, 4 weeks after stimulation, and 8 weeks after stimulation.<br>The change in S-IADL scores between baseline, 4 weeks after stimulation, and 8 weeks after stimulation.<br>Functional network changes in resting fMRI at 4 weeks after stimulation.<br>Change in FA values in DTI analysis at 4 weeks after stimulation.
			<Comparison between rTMS and Sham Groups><br>- The changes in scores of K-MMSE, K-MoCA, CDR, ADAS-Cog, COWAT, Stroop, TMT/PRM, RVP, and SWM between the rTMS treatment group and the sham group are compared.<br>- The changes in FA values in MRI DTI analysis between the rTMS treatment group and the sham group are compared.<br>- Qualitative comparison of functional network changes in fMRI between the rTMS treatment group and the sham

			group.
	Safety evaluation	- vital sign<br>- adverse effect	
Evaluation	Independent Assessment by Three Clinical Trial Investigators (Neuropsychologists)		

1. Clinical Trial Title

The effects of transcranial magnetic stimulation (ALTMS) on cognition in the patients with Alzheimer's disease: A single centered, randomized, evaluator-blinded, exploratory clinical trial

2. Clinical Trial institution

Name	location	telephone	fax
Samsung Medical Center	81, Ilwon-ro, Gangnam-gu, Seoul, Korea	02-3410-2378	02-3410-0052

3. Principal Investigator, collaborator, and Coordinator

■ **Principal investigator**

Name	Institution	Department	Position
Hyemin Jang	Samsung Medical Center	Neurology	Professor

■ **Collaborators and coordinator**

Name	Institution	Department	Position
Duk Lyul Na	Samsung Medical Center	Neurology	Professor
Young Min Sohn	Samsung Medical Center	Neurology	Professor
Sungshin Kim	Hanyang University	School of engineering	Professor
Young Hee Jung	Myongji Hospital	Neurology	Professor
Seung Hee Ahn	Samsung Medical Center	Neurology	Researcher
Eunhye Jo	Samsung Medical Center	Neurology	Researcher
Dayoung Ahn	Samsung Medical Center	Neurology	researcher

24hr contact information: Seung Hee Ahn (02-2008-4342)

4. Medical Device Manager

Name	Institution	Department	Position	Telephone
Won kyu Kim	Samsung medical center	Medical device division	Senior	02-3410-3239
Kyong bok kim	Samsung medical center	neurology	reseacher	010-2327-4038

5. Clinical Trial Sponsor

Name	Institution	Department	Position	Telephone
Hyemin Jang	Samsung Medical Center	Neurology	Professor	02-3410-2378

6. Objective and Background

A. Objectives

This clinical trial aims to demonstrate the improvement of memory and cognitive function using transcranial magnetic stimulation applied to the parietal region in patients with Alzheimer's disease.

1) Primary objective

● Comparison Before and After Stimulation in the rTMS Group:

-The change in ADAS-Cog scores at the 8-week follow-up after 4 weeks of repetitive Transcranial Magnetic Stimulation (rTMS) applied to the parietal region was measured.

2) Secondary objective

● Comparison Before and After Stimulation in the rTMS Group:

-The change in CANTAB PRM, RVP, and SWM scores at 4 and 8 weeks after 4 weeks of repetitive Transcranial Magnetic Stimulation (rTMS) applied to the parietal region was measured to assess cognitive function changes.

-The change in K-MMSE, K-MoCA, CDR-SOB, COWAT, Stroop, and TMT scores at 4 and 8 weeks after 4 weeks of rTMS treatment was measured and compared with baseline scores to assess cognitive function changes.

-The change in ADAS-Cog scores at 4 weeks after 4 weeks of rTMS treatment was measured and compared with baseline scores to assess cognitive function changes.

-The change in K-GDS short form scores at 4 and 8 weeks after using rTMS was measured and compared with baseline scores to assess emotional changes.

-The change in S-IADL scores at 4 and 8 weeks after using rTMS was measured and compared with baseline scores to assess changes in daily life functions.

-The change in FA (fractional anisotropy) values through diffusion tensor image (DTI) analysis **at 4**

weeks after using rTMS was measured and compared with baseline to assess white matter microstructure changes.

-The qualitative assessment of functional network changes in resting-state fMRI at 4 weeks after using rTMS was conducted by comparing with baseline, involving correlation, coherence, and spatial grouping based on temporal similarities in BOLD images to analyze simultaneously activated brain regions.

●Comparison Between the Experimental Group and the Control Group (Sham Group):

- A comparison of changes in K-MMSE, K-MoCA, CDR, ADAS-Cog, COWAT, Stroop, TMT/PRM, RVP, and SWM scores between the rTMS stimulation group and the sham group.
- A comparison of changes in MRI DTI analysis, specifically FA values, between the rTMS stimulation group and the sham group.
- A qualitative comparison of functional network changes in fMRI between the rTMS stimulation group and the sham group, focusing on assessing alterations in connectivity patterns.

B. Backgrounds

In South Korea, according to data from the Ministry of Health and Welfare in 2009, the elderly population aged 75 or above was already reported as 7.2% in the year 2000, marking the entry into an "aging society". By 2018, this percentage had increased to 14.3%, indicating the transition to an "aged society". It is predicted that by around 2030, similar to advanced countries like the United States and Europe, the elderly population will surpass 23% of the total population, entering an "ultra-aged society". The prevalence of dementia increases with age, doubling approximately every 5 years after the age of 65. Thus, while the prevalence is around 2-3% in the age group of 65-69, it geometrically escalates to about 4-6% in the 70-74 age group, 8-12% in the 75-80 age group, and over 20% in those aged 80 and above.

The rapid increase in the elderly population is closely related to a surge in dementia patients, resulting in social and economic issues that have now become significant national concerns. Two common causes of dementia are vascular dementia and Alzheimer's disease. Vascular dementia is

linked to risk factors such as brain infarcts, hypertension, and hypercholesterolemia, which can be managed to prevent progression. However, Alzheimer's disease, in contrast, lacks medication or non-pharmacological treatment to effectively improve cognitive functions or halt its progression. Notable medications include cholinesterase inhibitors or memantine (N-methyl-D-aspartate receptor antagonists).

Transcranial Magnetic Stimulation (TMS) is a non-pharmacological treatment method that involves stimulating the scalp to enhance brain activity with safety and minimal side effects. It has long been recognized for effectively reducing symptoms of conditions like depression, anxiety, and addiction. In recent years, TMS has gained attention for its potential to enhance brain activity in normal aging individuals, with some reports suggesting temporary improvements in language abilities after stimulation in the frontal lobe of Alzheimer's patients. However, research on improving cognitive functions or preventing cognitive impairment in Alzheimer's disease patients through TMS is limited.

Among these methods, Repetitive Transcranial Magnetic Stimulation (rTMS) involves delivering repeated stimulations of the same intensity and frequency to a specific brain area. This procedure is known to modulate cortical excitability over a certain period. The pattern of change in cortical excitability is influenced by factors such as the intensity, frequency, and total number of stimulations. Frequency is particularly crucial, with studies indicating that 1Hz low-frequency stimulation generally inhibits cortical excitability, while frequencies above 5Hz increase it. Long-term depression and long-term potentiation are involved in excitability inhibition and enhancement, respectively, through these processes, inducing neural plasticity. Prior studies reporting improvements in memory primarily utilized high-frequency stimulation at 20Hz.

Therefore, this study aims to investigate whether repetitive transcranial magnetic stimulation (rTMS, 20Hz) can lead to cognitive changes, including memory enhancement, in elderly individuals with Alzheimer's disease.

7. Inclusion Criteria, Exclusion Criteria, and Sample Size Determination

A. Research Hypotheses

● Null Hypotheses:

-There will be no significant difference in cognitive abilities before and after receiving 4 weeks of rTMS stimulation.

-There will be no significant difference in cognitive abilities between the group that received 4 weeks of rTMS stimulation and the group that received sham stimulation.

● Alternative Hypotheses:

-There will be a significant difference in cognitive abilities before and after receiving 4 weeks of rTMS stimulation.

-There will be a significant difference in cognitive abilities between the group that received 4 weeks of rTMS stimulation and the group that received sham stimulation.

B. Inclusion Criteria

1) aged between 55 and 90 years

2) diagnosed as either mild cognitive impairment due to AD or mild AD dementia on the basis of National Institute on Aging–Alzheimer's Association criteria, with amyloid positivity determined by PET or CSF testing

3) Objective memory impairment as indicated by at least 2 standard deviations below the age- and education-adjusted mean in verbal language test (SVLT) or Rey-copy figure test (RCFT) delayed recall tests

4) ≥ 18 of Mini-Mental State Examination (MMSE) score

5) no history of epilepsy

6) normal in electroencephalography (EEG)

7) no myocardial infarcts or arrhythmia in electrocardiography (ECG)

8) those who are literate

9) those who voluntarily submitted written consents.

C. Exclusion Criteria

1) with other medical or surgical diseases that caused dementia

2) with serious medical conditions such as cancer, heart disease, and major surgical conditions

3) with severe white matter hyperintensities (WMH) on magnetic resonance image (MRI), which were defined as deep WMH ≥25 mm and periventricular WMH ≥10 mm;

4) who could not undergo MRI due to side effects related to contrast agents, or claustrophobia;

5) who have implanted medical devices that are susceptible to electronic disturbance, such as pacemaker, or detachable metallic materials (prosthetics, braces, cochlear implants etc.);

6) who have undergone cerebrovascular surgery, such as coiling clipping, and carotid artery stenting;

7) who have difficulty breathing when sitting;

8) who have lost consciousness for more than one hour due to causes other than general anesthesia;

9) who have been hospitalized with a head trauma;

10) who cannot read even with glasses due to reduced vision;

11) who have difficulty understanding conversations due to hearing impairment even with a hearing aid;

12) who have taken ototoxic drugs or have been exposed to loud noises

13) unsuitable for clinical trial participation judged by investigators

D. Sample Size Determination

●Target number of participants: 32 participants (sham group 16 and rTMS group 16)

The rationale for the number of participants is based on previous studies. Koch et al. (2008) conducted a study involving 14 Alzheimer's disease patients to investigate the relationship between transcranial magnetic stimulation and cognitive function. Additionally, Wang et al. (2014) reported results from a study with 16 patients showing improvement in associative memory through transcranial magnetic stimulation.

Based on these previous studies, this current study aims to explore the changes in memory and cognitive function by applying transcranial magnetic stimulation to individuals with Alzheimer's disease. This study is an exploratory investigation led by researchers, with 16 patients in each experimental and control group, totaling 32 patients, to assess the extent of cognitive function changes. This study serves as an exploratory study for setting the sample size and evaluating cognitive function changes for future confirmatory clinical trials. In these trials, the effects of transcranial magnetic stimulation will be examined in a larger population of Alzheimer's disease patients, and achieving this goal will require further research.

8. Clinical Trial Duration

Duration from IRB/MFDS approval to completion: 24 months

Participant recruitment period: 12 months

Clinical observation and trial execution period: 10 months

Statistical analysis and report writing period: 2 months.

9. Methods

A. Clinical Trial Design and Randomized Allocation

1) Primary Outcome Measure

1)Study Design: Single-arm design<br>2)Allocation: Random assignment<br>3)After 4 weeks of rTMS stimulation, changes in K-MMSE, K-MoCA, CDR-SOB, ADAS-Cog, COWAT, Stroop, TMT, RVP, SWM, PRM scores will be compared at an 8-week follow-up.<br>4) masking: Blinded assessment by evaluators

2) Secondary Outcome Measure

1) Study Design: Single-arm design<br>2) Allocation: Random assignment<br>3) -Compare changes in K-GDS short form and S-IADL scores between baseline, 4 weeks after rTMS stimulation, and 8 weeks after.<br>-Compare changes in K-MMSE, K-MoCA, CDR-SOB, ADAS-Cog, COWAT, Stroop, TMT, RVP,<br>-SWM, PRM scores between baseline and after 4 weeks of rTMS stimulation.<br>-Compare FA values in DTI analysis at 4 weeks after rTMS stimulation.<br>-Examine functional network changes in resting-state fMRI at 4 weeks after rTMS stimulation.<br>4) masking: Blinded assessment by evaluators

1)Study Design: Parallel design<br>2)Allocation: Random assignment<br>3) Intervention Group: rTMS application group<br>Control Group: Sham group (sham coil emits sound only, no rTMS stimulation)<br>4) -Compare K-MMSE, K-MoCA, CDR, ADAS-Cog, COWAT, Stroop, TMT, RVP, SWM, PRM scores between intervention and control groups after 4 weeks.<br>-Compare FA values in DTI analysis between intervention and control groups at 4 weeks after.<br>-Examine functional network changes in resting-state fMRI between intervention and control groups at 4 weeks after.<br>5) masking: Blinded assessment by evaluators

B. Trial Procedures

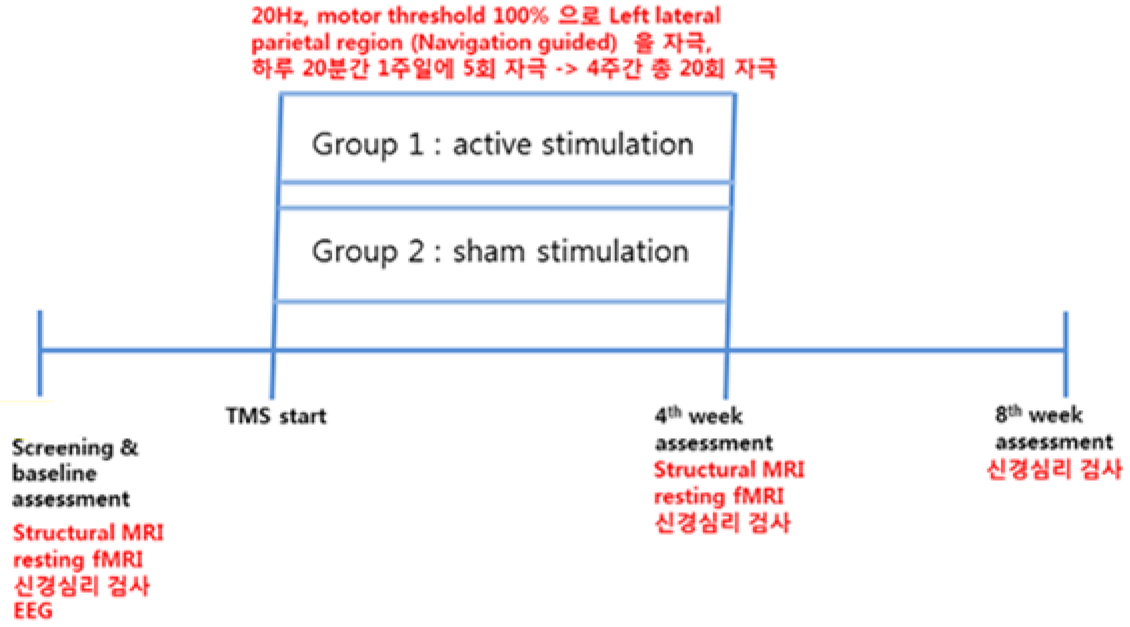

The study will include two groups:

- rTMS group: Actual rTMS stimulation will be administered for 4 weeks.
- Sham group: Sham stimulation (with sham coil producing sound only, no actual rTMS stimulation) will be administered for 4 weeks.

A total of 32 patients will be enrolled. They will be randomly divided into two groups:

- rTMS group (n=16): Patients receiving actual rTMS stimulation.
- Sham group (n=16): Patients receiving sham stimulation.

Both types of stimulation will be conducted for a duration of 4 weeks in all 32 patients.

C. Randomized Allocations

Randomization will be performed using a random block design generated with R programming. The randomization list will be created to allocate participants into different groups. The randomization allocator will match the assigned codes with the respective treatment settings for the experimental and control medical devices, which will then be provided to the researchers. The

researchers will administer stimulation to the participants in the order of their registration, following the randomized mode of allocation.

D. Masking

In this clinical trial, "blinding of evaluators" will be maintained.

① Participants: The stimulation coil with 8-shaped design is capable of mode changes. In the sham mode, a sound similar to the sound of the magnetic field being generated will occur, but no actual magnetic field will be generated. In the active mode, a real magnetic field will be generated, providing rTMS stimulation. The appearance of the device, sound, and the time of applying the rTMS coil are the same. Participants who are receiving rTMS stimulation for the first time may find it difficult to discern whether they are receiving active or sham stimulation. However, participants who have experienced the trial device at least once before may feel sensations or discomfort when receiving active stimulation, making complete blinding challenging.

② Operator: The individual delivering the rTMS stimulation will select either the sham or active mode from the coil, making them unblinded.

③ Investigator (Principal Investigator): The investigator enrolls patients who meet the criteria and are willing to participate in the study, as well as verifies patient conditions during outpatient visits. However, they will remain blind to which group the patients are assigned to.

④ Evaluator: Three independent assessors will evaluate cognitive function. These assessors will be blinded to which group the patients are assigned to.

⑤ Emergency Unblinding Procedure: In case of severe medical emergencies on a case-by-case basis, unblinding by the investigator might be considered. If the trial responsible person deems unblinding necessary, it will be performed. In such cases, the trial responsible person will ensure records of the unblinding procedure are maintained.

E. Stimulation Protocol

-Stimulation Frequency: 20 Hz, delivering stimulation at 20 Hz for 2 seconds in each train, followed by a 28-second rest period. A single stimulation site will receive a total of 40 trains (a total of 1600 pulses) in one session.

Stimulation Intensity: 100% of the motor threshold (MT), which can be decreased to 70% of the motor threshold in case of pain. Motor threshold is determined by stimulating the contralateral

motor cortex with TMS for 10 trials while the target muscle (Abductor pollicis brevis) is at rest. The lowest intensity that elicits visible muscle contractions in the target muscle for 5 out of 10 trials is selected as the motor threshold. This measurement will remain constant throughout the 4-week stimulation period.

-Stimulation Site: The stimulation site will be determined based on the resting functional MRI analysis of the baseline scan. The left lateral parietal region with the highest connectivity to the hippocampus will be selected for stimulation. During the 4-week stimulation period, an individualized medical guide will be used to fix the TMS transducer on the patient's scalp for accurate and consistent targeting. This medical guide will be produced using 3D printing based on the patient's brain MRI information, ensuring a customized fit for each patient.

-Duration: More than 20 minutes per session. Stimulation Period: Stimulation will be administered 5 times per week (Monday to Friday) for a total of 20 sessions over a 4-week period.

<Reference>

In a 2018 Neuroimage paper, Giacomo Koch reported a study employing a similar approach to the present research. He administered high-frequency rTMS stimulation at 20Hz with an intensity of 100% of the motor threshold for a total of 1600 pulses over a duration of 20 minutes. The stimulation was conducted five times a week (Monday to Friday) for a period of two weeks, followed by a two-week washout period, after which sham stimulation was administered for another two weeks, utilizing a cross-over design. Additionally, Koch mentioned that rTMS has already been used for prolonged periods (several months or years) as a clinical treatment method for depression patients.

Furthermore, in this study, an assessment is conducted immediately after administering the rTMS stimulation, followed by a 4-week break, and then another assessment at the 8-week mark. This approach was chosen because historically, the effects of rTMS were believed to be temporary, leading to evaluations primarily right after the stimulation. However, for this study, assessments at both immediate and 8-week intervals were included to investigate not only the immediate effects but also the potential long-term effects of the stimulation.

F. Concomitant Therapy

The provided information outlines the guidelines for concomitant therapy and medication use

during the clinical trial for rTMS (repetitive Transcranial Magnetic Stimulation) in Alzheimer's disease:

Cholinesterase Inhibitors and Memantine: Participants who are already on standard treatments for Alzheimer's disease, such as cholinesterase inhibitors (e.g., donepezil, rivastigmine) or memantine, can continue taking these medications during the rTMS treatment period.

Benzodiazepines and Seizure Threshold-Lowering Medications: Medications from the benzodiazepine class or any other drugs that can lower the seizure threshold are prohibited from being used concomitantly with the rTMS treatment.

Impact on Device Performance: The use of any other medical devices during the clinical trial period that might affect the efficacy, safety, or performance of the investigational medical device (rTMS device) is not allowed.

However, in certain unavoidable circumstances, if other medical devices need to be used due to the participant's treatment and ethical considerations, detailed records of their usage (device name, purpose, duration, etc.) should be maintained. These instances might lead to exclusion from compliance group analysis, ensuring that the scientific integrity of the trial is maintained.

This approach is designed to ensure the consistency and reliability of the trial results by controlling for the potential confounding effects of concomitant therapies and medications. It also adheres to ethical considerations for participant safety and welfare during the trial.

10. Observation Items and Observation Methods

Clinical trial schedule (for rTMS group)

Visit No			VISIT 1~3		VISIT 4~ VISIT 23	VISIT 24~25	VISIT 26
Visit schedule[1)]			Screening	baseline	rTMS application	Follow up	Follow up
					20 session for 4 weeks (Mon~Fri)	4 week	8 week
Visit window						± 7 day	± 7 day
Clinical trial explanation and informed consent preparation			●	(●)			
Eligibility determination (inclusion /exclusion criteria)			●	(●)			
Demographic data[2)]			●	(●)			
Vital sign[3)]			●	(●)		●	●
history taking [4)]			●	(●)			
Concomitant therapy[5)]			●	●			
Baseline EEG[13)]			●				
Baseline ECG[14)]			●				
Randomized allocation[6)]				●			
Medical device application (rTMS gruop/ Sham group[7)])					●		
Primary outcome	neuropsychological test [8)]		●			●	●
Secondary outcome	K-GDS short form, S-IADL[9)]		●			●	●
	MRI	Structural MRI	●			●	
		Diffusion tensor image[10)]	●			●	
		Resting Fmri[10)]	●*			●	

Verification of adverse event [11)		●	●[12)]	●	●

*** Neuropsychological assessments and various tests can be conducted during Screening or Baseline, depending on the situation, to ensure smooth progress.

1) Screening and Baseline assessments can be conducted on the same day, and if assessments or procedures are marked with (●), there is no need to repeat them during Baseline if they were performed during Screening.
2) The subject's initials, age, gender, alcohol/smoking status, education level, residence, and occupation will be assessed.
3) Blood pressure, heart rate, and body temperature will be measured.
4) Past and current medical history within the last 5 years will be collected based on the date of obtaining informed consent.
5) Medications currently taken or administered within the last 3 months from the date of obtaining informed consent will be investigated.
   - Cholinesterase inhibitors such as donepezil and memantine, commonly used in standard treatment for mild cognitive impairment or dementia, will continue to be taken during rTMS stimulation

6) The eligible participants for this clinical trial will undergo randomization as outlined below for enrollment.

- rTMS group: rTMS for 4 weeks

- sham group: Sham stimulation (using a sham coil producing only sound without actual rTMS stimulation) will be administered for a duration of 4 weeks.

7) Neuropsychological evaluation include the following:
   - General cognitive function: K-MMSE, ADAS-Cog, CDR, K-MoCA,
   - Attention & memory: CANTAB SVLT, RCFT
   - Frontal/executive test: COWAT, TMT, Stroop, CANTAB

However, the SVLT and RCFT tests will be administered only at the screening stage, and if the test results are available within 3 months from the date of obtaining informed consent, those results will be used for substitution. For the neurocognitive assessments conducted at the baseline (including CANTAB), if the test results are available within 6 months prior to the first rTMS stimulation, those results will be used for substitution.

9) – activity of daily living: S-IADL

- Depression: K-GDS short form

10) The acquisition of Diffusion Tensor Image and resting fMRI will be carried out simultaneously with MRI imaging (i.e., during the MRI scan). This means that any side effects associated with MRI will also apply to the acquisition of diffusion tensor image and resting fMRI. A portion of the 30-minute MRI scan will be dedicated to acquiring the diffusion tensor image and resting fMRI data. The information obtained from the fMRI will be used to create a customized 3D-printed guide for accurately positioning the TMS transducer on the patient's head for targeted treatment. This guide will ensure precise guidance for the treatment location based on the patient's individual data.

11) Any newly occurring symptoms or signs after rTMS application, as well as any worsening of underlying conditions, will be documented.

12) The presence of any adverse events will be assessed after each of the 20 sessions of rTMS stimulation.

13) If an EEG (electroencephalogram) test has been conducted within the past 6 months before the enrollment date, the results of that test can be collected and utilized as a replacement for the current study's EEG assessment.

14) If an ECG (electrocardiogram) test has been conducted within the past 6 months before the enrollment date, the results of that test can be collected and utilized as a replacement for the current study's ECG assessment.

A. Observation Items and Methods

(1) Clinical trial explanation and informed consent preparation (visit1)

Prior to entering the clinical trial, the purpose and content of this study are explained in detail to the participants or their legal guardians. The original signed informed consent form should be kept by the participant, and a copy should be provided to the trial subject or their legal guardian. The date of signature and the actual date of signing must be accurately recorded.

(2) inclusion/exclusion criteria (visit1)

To ensure the eligibility of participants for this trial, the investigator or their delegate must review all inclusion and exclusion criteria.

(3) demographic data and vital sign (visit 1)

Demographic information including subject initials, age, gender, alcohol/smoking status, education level, residence, and occupation will be collected. Evaluation of systolic and diastolic blood pressure, heart rate, and body temperature will also be conducted.

(4) Past medical history and concomitant medication (visit 1)

The medical history and medication history are collected through interviews and review of past medical records. The following information should be included in the medical history assessment. The past medical history and current medical conditions, including the occurrence timeline, treatment details, and progression status, of diseases such as stroke, mental disorders, diabetes, hypertension, and other relevant medical conditions that need to be screened are recorded. The medical history from the past 5 years up to the date of consent acquisition is investigated.

The concomitant medications are investigated for drugs taken or administered within the past 3 months up to the date of consent acquisition. The components, usage, dosage, duration, and purpose of the concomitant medications are verified.

Mild cognitive impairment or dementia standard treatments such as cholinesterase inhibitors and memantine can be continued during rTMS treatment.

(5) Neuropsychological assessments (Screening: Visit 2 / Evaluation: Visit 24-25 (Evaluation may be conducted in a single session or in some cases, in two sessions), Visit 26)

A. General cognitive function

① Alzheimer’s Disease Assessment Scale-Cognitive subscale (ADAS-Cog)

- The ADAS-Cog (Alzheimer's Disease Assessment Scale-Cognitive Subscale) is a

comprehensive assessment tool that evaluates cognitive functions such as memory, orientation, and language abilities. It is commonly used for assessing the efficacy of dementia treatments. The scores range from 0 to 85, with higher scores indicating more severe cognitive impairment.

- Memory: Immediate word recall, delayed word recall, word recognition, test instruction memory, delayed word recall
- Language Ability: Naming objects or fingers, difficulty in finding words, following commands, language expression, language comprehension.
- Executive Function: Constructive performance, conceptual performance, number cancellation

② Korean version of Mini-mental state examination (K-K-MMSE)

It is a comprehensive cognitive assessment that evaluates memory, spatial abilities, language, and other cognitive functions, with a total score of 30 points.

Evaluation items	score
Time orientation	5
Place orienatation	5
Registration	3
Recall	3
Attention and calculation	5
Language and visuospatial	9
Total	30

There is a cut-off value for considering dementia as being below 24 points, with a sensitivity of 92% and specificity of 91.5%. [11]

This assessment is conducted at the screening point to evaluate performance above 18 points. If the score is below 18 points, individuals are excluded from this clinical trial as they may have at least moderate dementia.[10] Furthermore, if there are test results within 3 months prior to the consent acquisition date, repeated testing will not be conducted for participation in this clinical trial.

We will compare the scores obtained during the screening conducted at 4 weeks and 8 weeks to assess if there are any differences, and also compare the differences between the sham group and

the stimulation group.

③ Clinical. Dementia Rating-sum of boxes (CDR-SOB)

The scores for memory, orientation, judgment and problem solving, community affairs, home and hobbies, and personal care and body care evaluations are combined in each respective domain to obtain a composite score ranging from 0 to 3. The CDR-SOB (Sum of Boxes) is calculated by summing up the scores from each domain.

Composite score	symptoms
0	normal
0.5	uncertain
1	mild
2	moderate
3	severe

④ Korean Montreal Cognitive Assessment (K-MoCA)

The MoCA, adapted and standardized considering Korean culture and linguistic characteristics, is an assessment tool designed to quickly screen for mild cognitive impairment. The subdomains include attention and concentration, executive functions, memory, language abilities, visuospatial skills, conceptual thinking, calculation abilities, and orientation. The total score is 30 points.

While K-MMSE mainly assesses language and memory, it often lacks proper evaluation of frontal lobe functions. In contrast, K-MoCA evaluates frontal lobe functions effectively, making it a complementary assessment tool to K-MMSE.

B. Attention & Memory

① Cambridge Neuropsychological Test Automated Battery (CANTAB)

A touchscreen-based cognitive assessment system primarily evaluates cognitive functions such as

memory, judgment, and attention using non-verbal stimuli through a computer interface.

The sequence of CANTAB is as follows :MOT→ DMS → RVP → PRM → RTI →SWM → PAL .

I. Motor Screening (MOT)

: This test involves a flashing "X" on a computer screen that requires participants to touch the "X" with their finger whenever it appears. The test is conducted a total of 13 times, with the initial 3 times serving as practice sessions. The final 10 trials are used for evaluation purposes.

II. Delayed Matching to Sample (DMS)

: This item evaluates visual memory and takes approximately 10 minutes to complete.

A picture appears in the box on the computer screen, and below it, there are 4 options. Remember the color and shape of the picture in the upper box, then choose the same picture from the options below. This task is conducted a total of 23 times, with 3 practice trials and 20 evaluation trials.

III. Rapid Visual Information Processing (RVP)

: This test evaluates attention and takes approximately 7 minutes to complete.

In this test, a square box on the computer screen displays numbers from 1 to 9 repeatedly. When a predetermined sequence of three numbers appears in consecutive order, press the button.

IV. Pattern Recognition Memory (PRM)

: This task assesses visual memory and takes approximately 5 minutes to complete.

A square box appears in the center of the screen, and various images are presented one by one inside that box. Your task is to remember which image you saw and then select the previously displayed image from the options provided.

V. Reaction Time (RTI)

The test takes 5 minutes. While holding down the right button on the press pad with your dominant hand, a yellow circle will flash and appear inside a white circle. When this happens, release the press pad button and quickly press the spot where the yellow circle appeared.

VI. Spatial Working Memory (SWM)

: This test evaluates executive function and takes 8 minutes. When you press the square, it will either

be empty or contain a blue square. If a blue square appears, fill in the black bar below it.

VII. Paired Associates Learning (PAL)

: This assesses visual memory and takes 10 minutes. When 6 white boxes appear on the screen, two of them will contain different pictures. Remember which picture appeared in which box. Later, a picture will be shown in the center of the screen, and you need to select the box where that picture was originally shown. There are no practice trials, and you will do this a total of 5 times. As the rounds progress, the number of pictures increases. You can go up to a maximum of 6 rounds, but if you fail more than 6 times, the test will end for that round.

② SVLT (Seoul Verbal Leaning test) and RCFT(Rey Complex Figure test)

The Seoul Verbal Learning Test and Rey Complex Figure Drawing Test will be conducted to assess the suitability for participation in this clinical trial. If the test results from within 3 months of the date of consent acquisition are available, there is no need to repeat the tests.

C. Frontal/executive test

① COWAT (Controlled Oral Word Association Test)

This test evaluates how many words a participant can voluntarily speak within 60 seconds that belong to given semantic categories (animals, supermarket) and phonemic categories (ㄱ, ㅇ, ㅅ) or start with the provided phonemes. This test is a representative assessment of frontal lobe functions.

·animal

·supermarket

·phonemic (ㄱ, ㅇ, ㅅ)

② Stroop test

This test assesses how reaction time varies based on attention and inhibitory abilities. For instance, if the word "빨강" (meaning red) is printed in yellow rather than red, participants need to inhibit their automatic reading response and instead name the color. This test evaluates inhibitory control and attention.

③ Trail making test (TMT)

The Trail Making Test (TMT) is an assessment where scattered numbers and days of the week on a sheet of paper need to be connected in order. TMT-A involves connecting randomly arranged numbers from 1 to 15 in the correct order, while TMT-B requires alternating connections between numbers 1 to 8 and days of the week "월" to "일". This test evaluates cognitive flexibility, attention, and visual-motor coordination.

④ Cambridge Neuropsychological Test Automated Battery (CANTAB)

(6) Korean version of Geriatric Depression Scale (K-GDS) short form

The Geriatric Depression Scale (GDS) short form consists of 15 questions. The highest score indicating very severe depression is 15 points, with a cut-off point at 8 points. This assessment is used to evaluate the presence and severity of depression in geriatric populations. [12]

Depression is one of the most common behavioral symptoms seen in Alzheimer's disease. Onset of depression in later life could be a precursor or a significant risk factor for Alzheimer's disease. When depression co-occurs with Alzheimer's disease, patients may experience more severe impairment in aspects such as their own quality of life, daily functioning, and physical aggression compared to cases without depression. This suggests a worse prognosis when depression is present alongside Alzheimer's disease.[13]

(7) S-IADL (Seoul-Instrumental Activities of Daily Living score)

Instrumental Activities of Daily Living (IADL) is a tool used to assess more complex functions necessary for older adults to maintain independent living. It evaluates abilities such as using the telephone, going out or traveling, shopping, meal preparation, household chores, manual tasks (sewing or nailing), laundry, proper medication management, and financial management.

(8) Electroencephalogram (EEG)

It records the brain's electrical activity in terms of frequency and amplitude, represented as an electroencephalogram (EEG). By analyzing the location of the electrical activity and the waveform of the brainwaves, it can diagnose abnormal electrical states within the brain and assess the degree

of arousal and brain activity in healthy individuals. In this study, resting-state EEG will be measured for 7 minutes each under conditions of eye closed and eye open.

(9) ECG

It is a test to determine the presence of heart disease, with a duration of approximately 10 minutes per test session.

(10) DTI (diffusion tensor image) (visit 3, visit 26)

The fractional anisotropy (FA) value, which reflects the movement of water molecules in white matter, is a measure of the integrity of white matter. By measuring the FA values of white matter connecting memory networks, it can be observed that these values are lower in early dementia or mild cognitive impairment compared to normal conditions. The hypothesis is that through transcranial magnetic stimulation (TMS), these values will increase, and this will be investigated in the study.

(11) resting functional MRI (visit 3, visit 26)

After administering TMS stimulation, changes in the functional connectivity of the memory network between the hippocampus and the cortex will be qualitatively compared using oxygen-dependent images (blood-oxygen-level-dependent images, BOLD) through resting-state fMRI analysis. In early dementia or mild cognitive impairment, functional connectivity is often reduced. The hypothesis is that through transcranial magnetic stimulation, the connectivity of the memory network will be enhanced, and this will be investigated in the study.

(12) Randomization and Participant Enrollment Number Assignment

Participants who consent to participate in the clinical trial and meet the inclusion/exclusion criteria will be enrolled, and randomization will be conducted. They will be randomly assigned to two groups and assigned a code. The clinical trial medical device will then be applied to the participants in the order of their enrollment.

- rTMS group: rTMS for 4 weeks

- sham group: Sham stimulation (using a sham coil producing only sound without actual rTMS stimulation) will be administered for a duration of 4 weeks.

Randomly assigned participants will be assigned participant registration numbers as follows:

Registration Number: SMC-Year Code-Trial Serial Number-Sequential Number

(13) rTMS treatment

For a duration of 4 weeks, the investigational medical device will be applied by the medical staff once a day for 20 minutes each session. Additionally, the medical staff responsible for administering the stimulation will create a log after each session. The subject log will include the application date, application status, start time of application, and any adverse reactions if they occur.

(14) verification of adverse effect

To gather information about any adverse events, subjects will be encouraged to report voluntarily, and the investigator will regularly interview them during scheduled or additional visits. Through interviews, questionnaires, and medical assessments, the investigator will assess the occurrence and details of any adverse events. Adverse events will be documented if there are new symptoms/signs that emerge after the application of the investigational medical device or if there is a worsening of underlying conditions. The adverse event record will include the onset and resolution dates, severity, extent, outcomes, actions taken related to the investigational medical device, causality assessment, treatment provided for the adverse event, determination of whether it qualifies as a serious adverse event or medical device adverse reaction, and relevant details. All of this information will be recorded in the case report form.

11. Predicted Side Effects and Precautions during use

The potential side effects that could occur during this clinical trial include:

11.1 repetitive Transcranial Magnetic Stimulatio (rTMS)[14]

(1) seizure

The prevalence of epileptic seizure among patients with epilepsys is approximately 1.4%, while it is less than 1% among healthy individuals. No adverse events have been reported within the range of stimulation indicated by the safety guidelines. Individuals with epilepsy or those with a history of epileptic discharges in pre-trial EEG assessments will be excluded from participation in the clinical trial.

(2) Transient acute hypomania induction

Although the possibility of occurrence is mentioned after stimulation of the left prefrontal area, there have been no reported cases in the specific left lateral parietal area targeted in this study.

(3) headache, neck pain, parathesia

It is an occasional side effect. If a patient complains of pain, the intensity will be lowered to 70% of the motor threshold. If the pain persists even at 70%, the stimulation will be stopped, and the participant will be excluded from the study.

(4) syncope

It can occur due to anxiety or psychological and physical discomfort. Differentiating it from seizures is necessary. Generally, if consciousness is quickly regained within a few seconds, or if there are prodromal symptoms such as dizziness, pallor, bradycardia, or nausea, it is often considered a vasovagal response rather than a seizure. If a loss of consciousness occurs, TMS stimulation should be immediately stopped. Medical personnel should assess airway and pulse, and if consciousness does not return for more than 30 seconds, appropriate measures should be taken similar to treating a seizure.

(5) transient hearling loss, tinnitus

During rTMS stimulation, earplugs will be used, and in case of any symptoms arising, an immediate referral to an otolaryngologist (ENT specialist) will be arranged. Participants with cochlear implants, history of significant noise exposure, or prior use of ototoxic medications will be excluded from the clinical trial.

Table. Reported side effects in high frequency rTMS [14]

Side effects	Frequency
Seizure	In patients with epilepsy~ 1.4%<br>In health individuals<1%
Transient acute hypomania induction	Possible occurrences during left prefrontal cortex stimulation.
syncope	Often appears due to psychological reasons rather than direct effects of rTMS.
Headache, localized pain, neck pain, parathesia	Frequent
Transient hearing loss	Possible
Burns	Occasionally reported

11.2 Electroencephalography (EEG)

There is no known risk associated with the EEG device currently used for diagnostic purposes at Samsung Medical Center.

11.3 MRI (structural MRI, resting fMRI)

There is no known risk associated with the device currently used for diagnostic purposes at Samsung Seoul Hospital, which is also known to be safe from causing claustrophobia or contrast agent-related side effects.

When anticipated side effects occur, they will be categorized into three levels based on their severity. The investigator will record the name of the side effect and its severity level in the case report form.

12. Termination or Withdrawal Criteria and Re-screening

12.1 Criteria for Study Termination and Handling Study Termination

12.1.1 Criteria for Study Termination

If serious adverse events occur during follow-up, these results will be discussed immediately by the clinical trial committee, and if the results are deemed sufficiently conclusive to justify termination of the study, the trial will be halted.

- If seizures or skin reactions occur during stimulation and the investigator deems it necessary to stop the stimulation, the trial will be halted.

- If the investigator deems it best for the participant to stop for any reason at any time, the trial will be halted.

Deviation from the protocol will not result in discontinuation of participants unless it poses a significant risk to their safety. Participants have the right to withdraw from the clinical trial voluntarily at any time. Participants who express the intention to discontinue their participation, fail to attend visits, or fail in follow-up observations for any reason may be considered as termination.

12.1.2 Handling of Study Termination

Interventions or treatments will be administered to discontinued participants as necessary, and their progress will continue to be observed.

All test results, the date of discontinuation, reasons for discontinuation, subsequent interventions, and progress after discontinuation will be documented in the case report form

In case the clinical trial is terminated, the Clinical Trial Investigator will notify the Institutional Review Board (IRB) in writing about the termination along with the reasons for termination.

12.2 Criteria for Withdrawal and Handling of Withdrawal

12.2.1 Criteria of Withdrawal

1) Patient's Request: Any patient who wishes to withdraw from the study can do so, but they will receive counseling from the medical staff about the need for long-term follow-up observation.

2) Serious, unexpected, or life-threatening adverse events requiring treatment discontinuation.

3) When the medical judgment of the healthcare provider indicates that further participation in the series of follow-up tests could be detrimental to the patient's health or well-being.

4) Failure to comply with the regulations outlined in the clinical trial protocol.

If a participant is suspected to have progressed to dementia during the course of the clinical trial and they do not refuse, their participation in the trial will continue. This does not constitute a criterion for exclusion from the trial. However, if acetylcholinesterase inhibitors or memantine are subsequently added for the participant's benefit, this information should be recorded in the concomitant medication section of the case report form.

Participants can voluntarily withdraw from the clinical trial at any time for any reason. Participants who express their intention to withdraw, fail to attend scheduled visits, or otherwise fail to comply with follow-up observations may be considered as having dropped out of the trial prematurely.

12.2.2 Handling of Withdrawal

- Participants who withdraw from the clinical trial due to simple mood changes will be excluded from the study population.

- If the discomfort is not due to simple mood changes but is caused by the trial itself, the underlying reason will be analyzed and attached to the case report.

12.3 Re-screening

12.3.1 Re-screening

Screening failure is defined as a situation where a trial participant has consented to participate in the clinical trial but cannot proceed with the trial due to reasons such as selection/exclusion criteria before randomization. Under the discretion of the Principal Investigator, screening failures can be re-screened based on the criteria outlined in the approved clinical trial protocol. Subjects who have been disqualified during the screening process may undergo re-screening only once, provided that

they provide their renewed written consent. Prior to re-screening, these subjects must provide a new informed consent, and they will be assigned a new subject identification number.

13. Outcome Measure, Statistical Analysis, and Interpretation

13.1. Outcome Measure

A. Primary Outcome Measure

Change in ADAS-Cog test scores after 8 weeks of rTMS stimulation and the difference in change scores at the 8-week time point.

B. Secondary Outcome Measure

Change in neuropsychological test scores at 4 weeks and 8 weeks after 4 weeks of rTMS stimulation, and the difference in change scores between the two groups.

the change in MRI measurements at the 4-week time point and the difference in change between the two groups

	Items	Outcome measures
Neuro-psychological test	CANTAB	Change in PRM (Pattern Recognition Memory), RVP (Rapid Visual Processing), and SWM (Spatial Working Memory) scores at 4 weeks and 8 weeks after intervention
	K-MMSE	Change in K-MMSE score at 4 weeks and 8 weeks after the intervention.
	K-MoCA	Change in K-MOCA score at 4 weeks and 8 weeks after the intervention.
	ADAS-Cog	Change in ADAS-cog score at 4 weeks after the intervention.
	CDR	Change in CDR-SOB score at 4 weeks and 8 weeks after the intervention.
	COWAT	Change in COWAR score at 4 weeks and 8 weeks after the intervention.
	Stroop	Change in stroop score at 4 weeks and 8 weeks after the intervention.
	TMT	Change in TMT score at 4 weeks and 8 weeks after the intervention.
	S-IADL	Change in SIADL score at 4 weeks and 8 weeks after the intervention.

	K-GDS short form	Change in K-GDS short form score at 4 weeks and 8 weeks after the intervention.
MRI	DTI	The change in fractional anisotropy (FA) values after 4 weeks
	Resting fMRI	A qualitative analysis using BOLD (blood-oxygen-level-dependent) images will be conducted after 4 weeks.

13.2. Statistical Analysis of the Efficacy

This study is an exploratory, investigator-driven clinical trial. Initially, instead of applying statistical analysis for clinical efficacy, the focus will be on exploring the differences in the changes of primary and secondary variables before and after the application of rTMS (or Sham coil) in the experimental and sham groups. The aim is to investigate how much difference exists and to describe these findings through exploratory research.

Additionally, for the statistical analysis of clinical efficacy, a linear mixed-effects model will be employed. This model will incorporate the intervention group, time points, and other key covariates as fixed effects. This approach will allow for the assessment of the intervention's impact over time while accounting for individual variations and potential confounding factors.

14. Criteria, Methods, and Reporting for Safety Evaluation, Including Adverse Events

All registered participants who underwent rTMS application in this study will be included in the safety evaluation. Participants who did not receive rTMS or for whom safety information was not collected during follow-up observations will be excluded from the analysis.

14.1 Criteria for Safety Evaluation

- Symptoms reported by the participant related to adverse events occurring during or after the use of the medical device.

Table. Reported side effects in high frequency rTMS [14]

Side effects	Frequency
Seizure	In patients with epilepsy~ 1.4%<br>In health individuals<1%
Transient acute hypomania induction	Possible occurrences during left prefrontal cortex stimulation.
syncope	Often appears due to psychological reasons rather than direct effects of rTMS.
Headache, localized pain, neck pain, paresthesia	Frequent
Transient hearing loss	Possible
Burns	Occasionally reported

- vital sign (blood pressure, body temperature, heart rate)

14.2 Method for Safety Evaluation

The safety is evaluated through adverse events and vital signs. All reported adverse events following rTMS application will be summarized in terms of severity and causality. The frequency and percentage of the number of participants and occurrences of adverse events will be presented in a chart. As for vital signs, continuous variables will be summarized using mean, standard deviation,

median, and range, while categorical variables will be summarized using frequency and percentage. Additionally, clinically significant results will be summarized and presented separately.

14.3 Reporting adverse events

14.3.1 Subjects of Evaluation

All participants involved in the clinical trial

14.3.2 Criteria and Methods

The clinical trial personnel are responsible for recording the symptoms, onset date, and resolution date of adverse reactions on the adverse reaction record form if adverse reactions occur in the participants during the clinical trial.

(1) Severity of Adverse Reactions<br>The clinical trial personnel should use the assessment criteria as a reference to indicate mild, moderate, severe, etc. on the adverse reaction record form.<br>(2) Causality<br>The clinical trial personnel should determine the causality according to the assessment criteria and indicate it on the adverse reaction record form.<br>(3) management of adverse reaction<br>In case there are changes in the clinical trial procedures such as modifications in usage methods, reduced frequency of use, or discontinuation due to adverse reactions, these changes should be documented on the adverse reaction record form.<br>(4) the result of management<br>It should be possible to record the resolution or attenuation of adverse reactions following interventions on the adverse reaction record form.<br>(5) opinion on adverse reaction<br>The opinion of the clinical coordinator, regarding adverse reactions will be documented.

15. Reference

1. Kim, S., et al., *Selective and coherent activity increases due to stimulation indicate functional distinctions between episodic memory networks.* Science Advances, 2018. **4**(8).
2. Nilakantan, A.S., et al., *Network-targeted stimulation engages neurobehavioral hallmarks of age-related memory decline.* 2019. **92**(20): p. e2349-e2354.
3. Cotelli, M., et al., *Effect of Transcranial Magnetic Stimulation on Action Naming in Patients With Alzheimer Disease.* Archives of Neurology, 2006. **63**(11): p. 1602-1604.
4. Cotelli, M., et al., *Improved language performance in Alzheimer disease following brain stimulation.* J Neurol Neurosurg Psychiatry, 2011. **82**(7): p. 794-7.
5. Cotelli, M., et al., *Transcranial magnetic stimulation improves naming in Alzheimer disease patients at different stages of cognitive decline.* European Journal of Neurology, 2008. **15**(12): p. 1286-1292.
6. Wang, J.X., et al., *Targeted enhancement of cortical-hippocampal brain networks and associative memory.* Science, 2014. **345**(6200): p. 1054-1057.
7. Koch, G., et al., *Transcranial magnetic stimulation of the precuneus enhances memory and neural activity in prodromal Alzheimer's disease.* Neuroimage, 2018. **169**: p. 302-311.
8. Ridding, M.C. and J.C. Rothwell, *Is there a future for therapeutic use of transcranial magnetic stimulation?* Nat Rev Neurosci, 2007. **8**(7): p. 559-67.
9. Bondi, M.W., et al., *Neuropsychological criteria for mild cognitive impairment improves diagnostic precision, biomarker associations, and progression rates.* Journal of Alzheimer's Disease, 2014. **42**(1): p. 275-289.
10. Weissman, M.M., et al., *Psychiatric disorders (DSM-III) and cognitive impairment among the elderly in a U.S. urban community.* Acta Psychiatr Scand, 1985. **71**(4): p. 366-79.
11. 강연욱, 나덕렬, and 한승혜, *치매환자-들을 대상으로 한 K-MM8E 의 타당도연구.* 1997.
12. 정인과, et al., *노인우울척도 (Geriatric Depression Scale) 의신뢰도, 타당도 연구.* J Korean Neuropsychiatr Assoc, 1997. **36**(1).
13. 곽용태, 양영순, and 구민성, *알츠하이머병에서의우울증.* 대한치매학회지, 2014. **13**: p. 27-36.
14. Rossi, S., et al., *Safety, ethical considerations, and application guidelines for the use of transcranial magnetic stimulation in clinical practice and research.* Clinical neurophysiology, 2009. **120**(12): p. 2008-2039.
