# Supplementary material for: Effectiveness of Personalized Hippocampal Network–Targeted Stimulation in Alzheimer Disease: A Randomized Clinical Trial
Source: JAMA Netw Open. 2024 May 6;7(5):e249220. doi: 10.1001/jamanetworkopen.2024.9220 (PMC11074813; doi:10.1001/jamanetworkopen.2024.9220)
Supplement: Supplement 3. — Data Sharing Statement [file jamanetwopen-e249220-s003.pdf]

# Data Sharing Statement

Jung. Effectiveness of Personalized Hippocampal Network–Targeted Stimulation in Alzheimer Disease. *JAMA Netw Open*. Published May 06, 2024.
doi:10.1001/jamanetworkopen.2024.9220

**Data**
**Data available:** Yes
**Data types:** Deidentified participant data
**How to access data:** dukna@naver.com
**When available:** beginning date: 12-01-2024

**Supporting Documents**
**Document types:** None

**Additional Information**
**Who can access the data:** researchers whose proposed use of the data has been approved
**Types of analyses:** for research
**Mechanisms of data availability:** researchers whose proposed use of the data has been approved
